# Single-cell characterization and quantification of translation-competent viral reservoirs in treated and untreated HIV infection

**DOI:** 10.1371/journal.ppat.1007619

**Published:** 2019-02-27

**Authors:** Marion Pardons, Amy E. Baxter, Marta Massanella, Amélie Pagliuzza, Rémi Fromentin, Caroline Dufour, Louise Leyre, Jean-Pierre Routy, Daniel E. Kaufmann, Nicolas Chomont

**Affiliations:** 1 Department of Microbiology, Infectiology and Immunology, Université de Montréal, Montreal, Quebec, Canada; 2 Centre de Recherche du Centre Hospitalier de l’Université de Montréal, Montreal, Quebec, Canada; 3 Division of Hematology & Chronic Viral Illness Service, McGill University Heath Centre, Montreal, Quebec, Canada; 4 Department of Medicine, Université de Montréal, Montreal, Quebec, Canada; University of North Carolina at Chapel Hill, UNITED STATES

## Abstract

The phenotypic characterization of the cells in which HIV persists during antiretroviral therapy (ART) remains technically challenging. We developed a simple flow cytometry-based assay to quantify and characterize infected cells producing HIV proteins during untreated and treated HIV infection. By combining two antibodies targeting the HIV capsid in a standard intracellular staining protocol, we demonstrate that p24-producing cells can be detected with high specificity and sensitivity in the blood from people living with HIV. In untreated individuals, the frequency of productively infected cells strongly correlated with plasma viral load. Infected cells preferentially displayed a transitional memory phenotype and were enriched in Th17, peripheral Tfh and regulatory T cells subsets. These cells also preferentially expressed activation markers (CD25, HLA-DR, Ki67), immune checkpoint molecules (PD-1, LAG-3, TIGIT, Tim-3) as well as the integrins α4β7 and α4β1. In virally suppressed individuals on ART, p24-producing cells were only detected upon stimulation (median frequency of 4.3 p24+ cells/10^6^ cells). These measures correlated with other assays assessing the size of the persistent reservoir including total and integrated HIV DNA, Tat/rev Induced Limiting Dilution Assay (TILDA) and quantitative viral outgrowth assay (QVOA). In ART-suppressed individuals, p24-producing cells preferentially displayed a transitional and effector memory phenotype, and expressed immune checkpoint molecules (PD-1, TIGIT) as well as the integrin α4β1. Remarkably, α4β1 was expressed by more than 70% of infected cells both in untreated and ART-suppressed individuals. Altogether, these results highlight a broad diversity in the phenotypes of HIV-infected cells in treated and untreated infection and suggest that strategies targeting multiple and phenotypically distinct cellular reservoirs will be needed to exert a significant impact on the size of the reservoir.

## Author summary

HIV persists in a small pool of infected CD4+ T cells during ART. A better characterization of these cells is a pre-requisite to the development of HIV eradication strategies. We developed a novel assay, named HIV-Flow, to simultaneously quantify and characterize reservoir cells in individuals receiving ART. With this assay, we found that a median of only 5 cells/million have the ability to produce the HIV protein Gag in individuals on suppressive ART. These frequencies correlated with other assays aimed at measuring HIV reservoirs. Importantly, we show that the HIV reservoir is phenotypically diverse, with numerous cell subsets contributing to the pool of persistently infected cells. Nonetheless, we identified several markers preferentially expressed at the surface or these rare reservoir cells, including immune checkpoint molecules and homing receptors. By combining these markers, we identified discrete cellular subsets highly enriched in HIV-infected cells. This novel assay will facilitate the identification of markers expressed by cellular HIV reservoirs.

## Introduction

The persistence of a small pool of infected CD4+ T cells displaying a memory phenotype is one of the major hurdles to HIV eradication [1–4]. This persistent viral reservoir is established during the early phase of infection and decays slowly over time [5–7]: more than 70 years of continuous ART would be required to completely purge this small pool of infected cells [8–10]. Therefore, additional therapeutic approaches specifically targeting persistently infected CD4+ T cells are needed to achieve a cure [11]. The development of such targeted strategies requires a deeper characterization of the nature of the cells in which HIV persists during prolonged ART.

A cellular marker that would identify HIV-infected cells with high specificity is still lacking. Although CD32a was originally proposed as a specific marker that highly enriches in HIV-infected cells in individuals on ART [12], recent studies challenged these findings [13–18]. Other cellular markers preferentially expressed by persistently infected cells have been reported, including CD30, CD2, PD-1, LAG-3, TIGIT and CTLA-4 [19–22]. However, these markers do not specifically identify infected cells and do not capture the entire pool of HIV reservoir cells. Similarly, several cell subsets were demonstrated to be enriched in HIV, including Tregs [23], Tfh cells [24, 25] and Th17 cells [26, 27].

Several assays have been developed to measure the frequency of infected cells in ART-suppressed individuals [28, 29]. Since the majority of viral genomes are defective [30], PCR-based assays measuring the frequencies of cells harboring HIV DNA overestimate the size of the replication-competent reservoir [31]. Of note, these defective proviruses are generated during the first rounds of viral replication following transmission, indicating that HIV DNA measurements may overestimate the size of the reservoir even in individuals who received ART during the first few weeks of infection [32]. Inducible RNA assays such as the Tat/rev Inducible Limiting Dilution Assay (TILDA) measure the frequency of cells harboring viral genomes that have the ability to produce viral transcripts upon maximal stimulation [29, 33, 34]. Nevertheless, these assays still overestimate the size of the replication-competent reservoir since a fraction of defective genomes retains the capacity to produce viral transcripts upon stimulation [35]. Other inducible assays measuring the frequency of cells that can produce viral particles after stimulation represent interesting alternatives. However, these assays also require limiting dilution cultures and multiple RNA extraction and RT-PCR, which make them both expensive and cumbersome [36, 37]. The quantitative viral outgrowth assay (QVOA) measures the frequency of resting CD4+ T cells harboring replication competent proviruses [1, 2, 38, 39] and is often considered the gold standard since it provides a minimal estimate of the size of the HIV reservoir. However, it is also time-consuming and labour intensive, and underestimates the size of the reservoir to various levels in different individuals [30, 31]. Finally, full-length sequencing of HIV genomes has the advantage of capturing intact non-induced genomes but does not functionally assess the ability of these intact viruses to be activated or to replicate in culture [30, 40].

All these methods offer complementary approaches to measure the magnitude of the HIV reservoir during ART. However, unless these assays are combined with cell sorting, none of them allow the phenotypic characterization of the cells in which HIV persists. Flow-cytometric approaches detecting viral proteins would allow to directly phenotype HIV-infected cells. Studies reporting detection of p24+ cells by flow cytometry in clinical samples [41, 42] may not exclusively capture HIV-infected cells due to the low specificity of antibodies targeting p24 [43–45]. Consistent with these findings, Graf et al. showed that cells expressing high levels of p24+ are highly enriched for HIV DNA, whereas cells expressing intermediate or low levels of p24 are more rarely infected [41]. Recently, fluorescent *in situ hybridization* (flow-FISH) approaches have been developed to detect viral transcripts in HIV-infected CD4+ T cells [14, 46, 47]. These flow cytometry-based assays enable the phenotypic characterization of HIV-infected cells expressing the *gag* transcript and/or Gag protein. Together, these studies reported complementary information on the nature of persistently infected cells, on the kinetics of viral reactivation from latency and on the ability of latency reversing agents (LRAs) to induce viral production from latent reservoirs.

Although these assays represent powerful novel technologies to quantify and characterize the cells in which HIV transcripts and/or proteins are produced, several aspects limit their use on a large-scale basis. First, they rely on *in situ* hybridization, and may consequently miss cells harboring viral variants (or HIV subtypes) in which probes may not optimally hybridize to RNA transcripts. Second, they require relatively large number of cells (10-20x10^6^ CD4+ T cells) due to material loss occurring during the multiple hybridization and amplification steps required to visualize HIV RNA transcripts by flow cytometry. Third, target-specific sets of probes remain expensive and RNA flow-FISH assays require 2–3 days to be completed.

Herein, we sought to develop a simplified alternative version of these assays that requires relatively small cell input (5-10x10^6^ CD4+ T cells) and can be used on a large-scale basis to characterize long-lived HIV reservoirs at the single cell level by flow cytometry. Due to the aforementioned limitations associated with the use of probes specific for RNA transcripts, we focused on the development of an assay that would detect HIV proteins with high specificity and sensitivity using a combination of antibodies.

## Results

### Detection of p24-producing cells in CD4+ T cells isolated from untreated and ART-suppressed HIV-infected individuals

**Fig 1:**
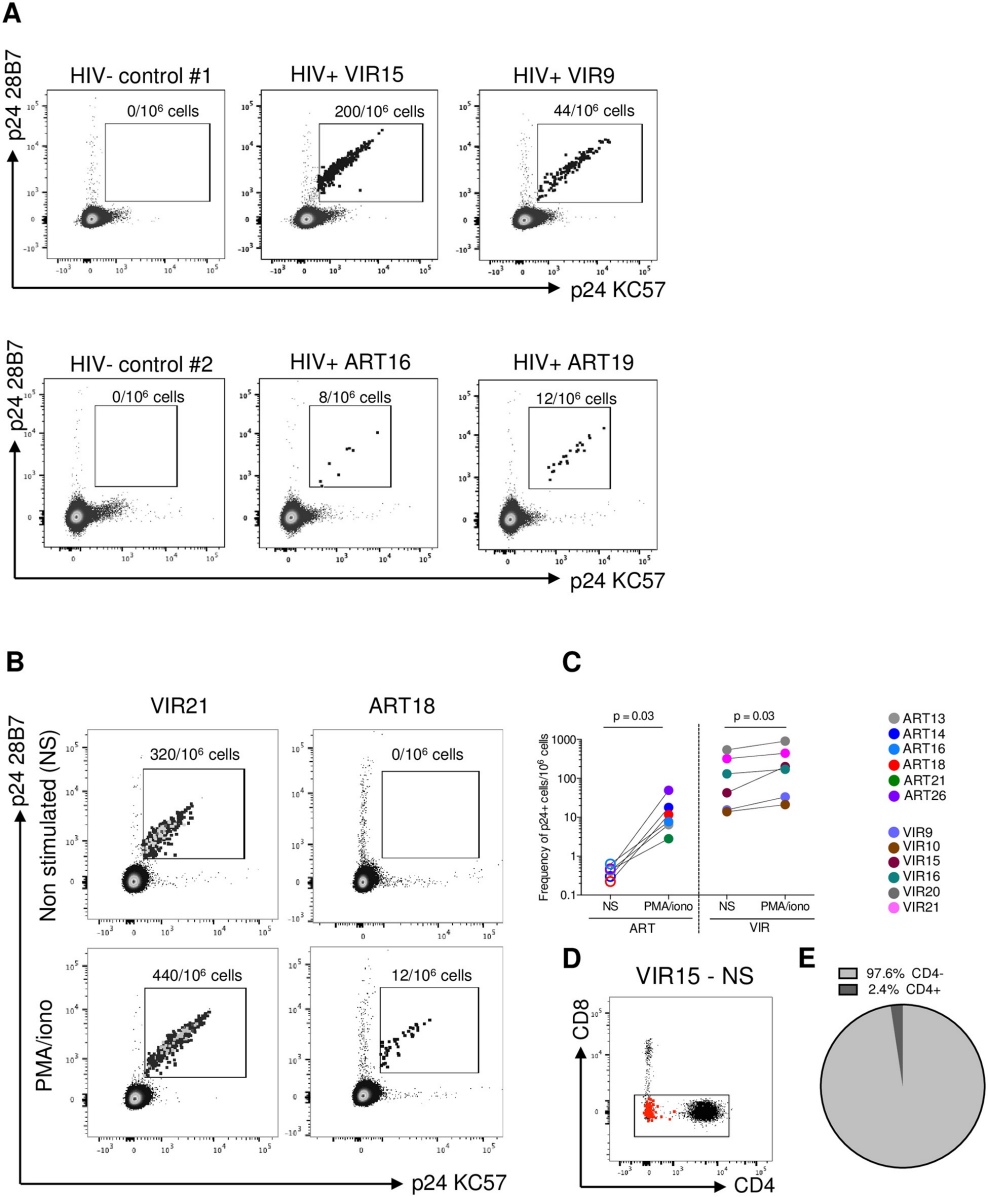
HIV-Flow allows the detection of p24-producing cells in samples from viremic and ART-suppressed individuals. (A) Representative dot plots showing the p24 KC57-PE/p24 28B7-APC co-staining in purified CD4+ T cells stimulated with PMA/ionomycin for 18h (viremic individuals) or 24h (ART-suppressed individuals). In each experiment, sample from an HIV-negative control was included. (B) Representative dot plots showing the detection of p24+ cells in the absence of stimulation (NS) or after PMA/ionomycin stimulation in samples from a viremic and an ART-suppressed individual. (C) Frequencies of p24+ cells in the absence of stimulation or following PMA/ionomycin stimulation in samples from 6 ART-suppressed (ART) and 6 viremic individuals (VIR). Each sample is represented by a unique color-coded symbol. Undetectable measures are represented as opened symbols, and limits of detection are plotted. For statistical analyses, Wilcoxon matched-pairs signed rank test was performed. (D) Representative dot plot showing cell surface CD4 expression in p24+ cells (represented as red dots) obtained from a viremic individual. (E) Pie chart representing the average proportions of p24+ cells expressing CD4 on their surface in samples from 6 viremic individuals.

The detection of p24+ cells by flow cytometry in clinical samples is technically challenging, since antibodies specific for HIV proteins are notorious for their limited specificity [43–45]. We hypothesized that combining antibodies targeting several viral epitopes would reduce the number of false positive events by improving the specificity of the staining. We used a combination of two antibodies targeting distinct epitopes of the HIV protein Gag (clones KC57 and 28B7, S1A Fig). To induce or enhance p24 production, all samples were stimulated with PMA/ionomycin. As expected, each antibody resulted in significant background when used individually in samples from HIV-uninfected individuals (S1B Fig). By combining these two antibodies, no signal was observed within the double positive gate with samples from HIV negative controls. In contrast, a clear double positive signal was obtained in samples from people with HIV (Fig 1A, S1 Fig).

We used this method (referred as HIV-Flow) to compare the frequency of cells producing p24 in samples obtained from treated and untreated individuals with HIV in the absence of stimulation or following stimulation (Fig 1B). Without stimulation, we failed to detect p24+ cells in samples obtained from six ART-suppressed individuals, whereas p24-producing cells were detected in samples from all six viremic participants (Fig 1C). Of note, the vast majority (97.6%) of these productively infected cells did not express CD4, likely as a consequence of the down-regulation of the receptor by Nef, Vpu and Env (Fig 1D and 1E) [48–50]. Stimulation with PMA/ionomycin revealed a population of p24-producing cells in all samples from the six virally suppressed individuals, which is consistent with the translationally inactive nature of the viral reservoir during ART (p = 0.03, Fig 1C). In addition, frequencies of p24-producing cells modestly increased in samples from the six untreated participants (2.1-fold, p = 0.03, Fig 1C), suggesting that translationally inactive infected cells are also present during untreated HIV infection. In addition to this slight increase in the frequency of p24+ cells, we observed a significant increase in the mean fluorescence intensity within the p24+ population for both antibodies (2.2 and 2.7-fold for p24 28B7 and p24 KC57, respectively, p = 0.03 for both, S2 Fig), indicating that the amount of p24 molecules per cell increased upon stimulation.

### Specificity, sensitivity, reproducibility and linearity of HIV-Flow

**Fig 2:**
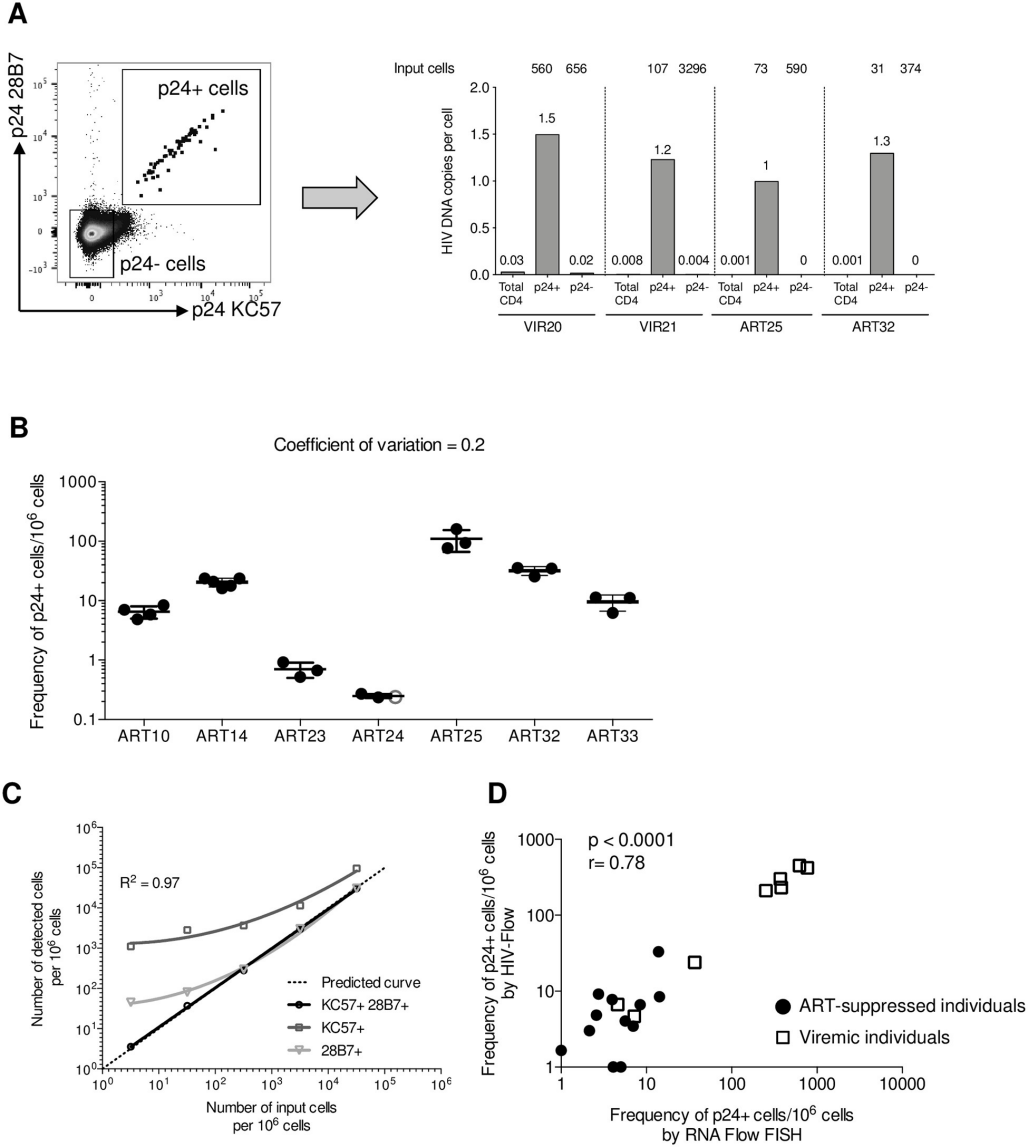
Specificity, sensitivity and reproducibility of HIV-Flow. (A) Representative dot plot showing the gating strategy used to sort p24+ and p24- cells following stimulation of CD4+ T cells with PMA/ionomycin (left). Total HIV DNA was quantified by ultrasensitive PCR in each sorted subset as well as in total unsorted CD4 T cells (right). (B) Repeated measures of the frequencies of p24+ cells from 7 ART-suppressed individuals in 2–5 independent experiments. Medians and interquartile ranges are shown. (C) Frequency of p24+ cells assessed by single staining p24 KC57 or p24 28B7, or by dual staining p24 KC57/p24 28B7 in *in vitro* infected CD4+ T cells spiked at different ratios in uninfected CD4+ T cells purified from an HIV uninfected individual. Predicted curve is represented by the dashed line. (D) Correlation between the frequency of p24+ cells assessed by HIV-Flow and the RNA Flow FISH assay. For statistical analyses, non-parametric Spearman correlation was performed.

To evaluate the specificity of the HIV-Flow assay, we used flow cytometry cell sorting to isolate p24+ and p24- cells following PMA/ionomycin stimulation of CD4+ T cells obtained from four individuals with HIV (two untreated and two suppressed on ART). Quantification of HIV DNA by ultrasensitive PCR in the sorted populations demonstrated a marked enrichment of HIV genomes in the p24+ cells compared to their negative counterparts (Fig 2A). p24+ cells harbored 1 to 1.5 HIV DNA copies per cell whereas 0 to 0.02 HIV genomes/cell were measured in the negative fractions (Fig 2A). An average 800-fold enrichment in cells harboring HIV DNA was observed in sorted p24+ cells compared to unsorted CD4+ T cells. Of note, single positive cells contained low levels of HIV DNA (S3 Fig).

To further validate the specificity of the assay, we sorted single p24- and p24+ cells from three ART-suppressed individuals and subjected these single cells to HIV DNA amplification (S4 Fig). The presence of a single cell per well was confirmed by the detection of CD3 gene. While almost all p24- cells were devoid of HIV DNA, HIV genomes were detected in 60% to 75% of the wells containing p24+ cells, indicating that the majority of the p24+ events detected by HIV-Flow harbored HIV DNA that could be amplified using our assay.

To evaluate the reproducibility of HIV-Flow, we measured the frequency of p24-producing cells upon stimulation with PMA/ionomycin in seven samples from virally suppressed individuals analyzed in 2–5 independent experiments (Fig 2B). The mean coefficient of variation was 0.2, indicating that HIV-Flow measures were reproducible. To assess the linearity of HIV-Flow measures, we spiked *in vitro* HIV-infected CD4+ T cells in uninfected CD4+ T cells isolated from an HIV-uninfected control (Fig 2C). The co-staining p24 KC57/p24 28B7 showed an excellent linearity to the lowest dilution tested (3.2 p24+ cells/10^6^ cells; R^2^ = 0.97). In contrast, each individual staining was weak predictor of the frequency of p24+ cells, confirming the low specificity of p24 antibodies when used individually [43–45]. To compare HIV-Flow and the mRNA Flow FISH assay for their ability to detect infected cells in clinical samples, we applied our method on samples previously analyzed by Baxter et al. [46]. We observed a highly significant correlation between both assays (r = 0.78, p<0.0001, Fig 2D), indicating that HIV-Flow and mRNA Flow FISH provide similar estimates of the size of the translation competent reservoir in clinical samples.

### Relationships between HIV-Flow, plasma viral load and reservoir measures

**Fig 3:**
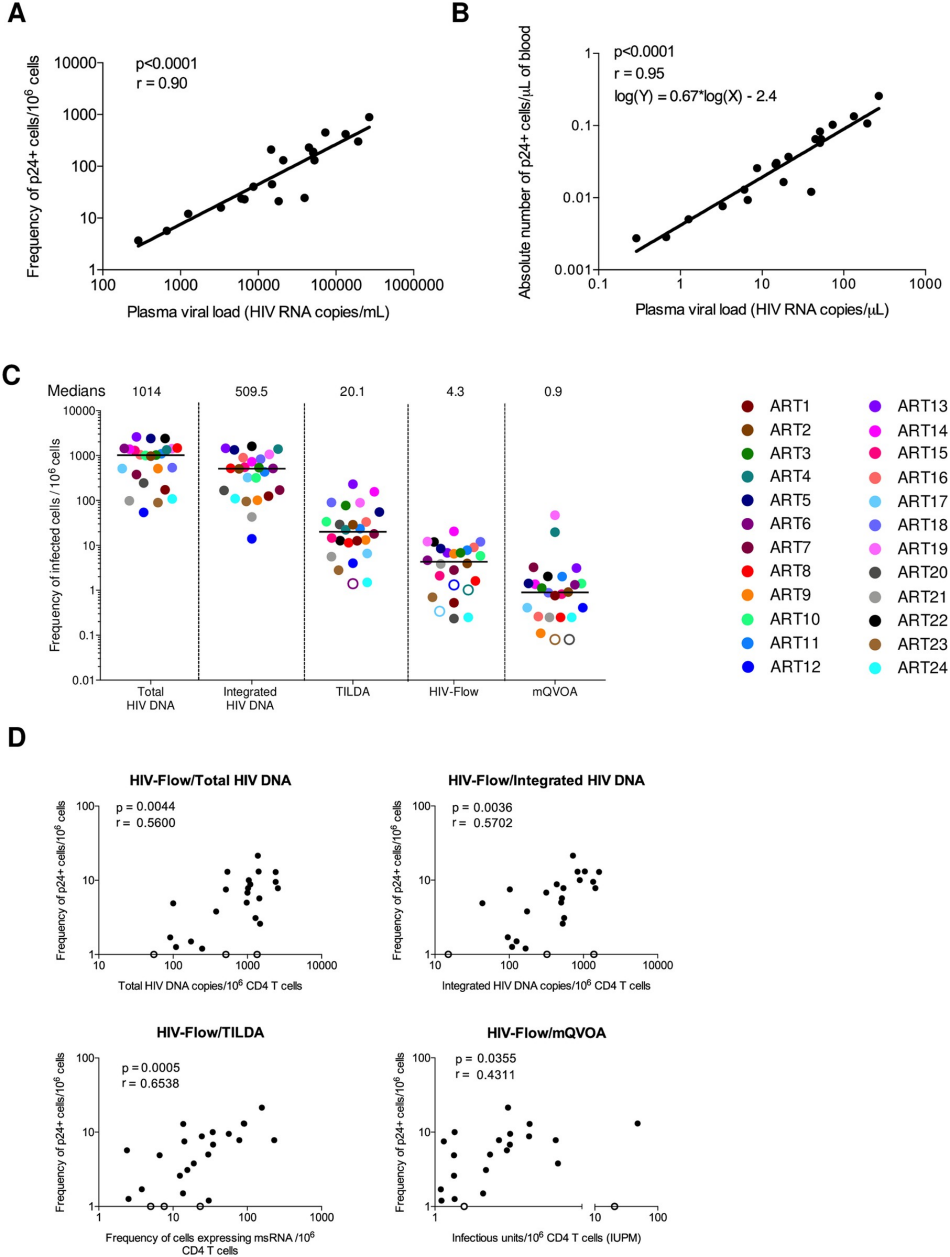
HIV-Flow correlates with other assays measuring the size of the HIV reservoir. (A) Correlation between plasma viral load and the frequency of p24+ cells in purified CD4 T cells from 20 chronically infected untreated individuals following stimulation with PMA/ionomycin. Data were log transformed and non-parametric Spearman correlation was used for statistical analyses. (B) Correlation between plasma viral load and the absolute number of p24+ cells per μl of blood. Data were log transformed and a linear regression was performed between plasma viral load (HIV RNA copies/μL) and the absolute numbers of p24-producing cells per μL of blood. (C) Frequencies of infected cells in samples from 24 ART-suppressed individuals were estimated using 5 different assays (total HIV DNA, integrated HIV DNA, TILDA, HIV-Flow, and mQVOA). Black lines represent medians. Each sample is represented by a unique color-coded symbol. Undetectable measurements are represented as open symbols, and limits of detection are plotted. (D) Correlations between the frequency of p24-producing cells assessed by HIV-Flow and other assays measuring the reservoirs size (total HIV DNA, integrated HIV DNA, TILDA and mQVOA). For statistical analyses, data were log transformed and non-parametric Spearman correlations were performed.

We used HIV-Flow to measure the frequency of p24-producing cells upon stimulation with PMA/ionomycin in CD4+ T cells isolated from 20 untreated and chronically infected individuals (Table 1, VIR1-20). We measured a median frequency of 87 p24+ cells/10^6^ cells (IQR = 22–215 cells/10^6^ cells). Frequencies of p24+ cells were strongly correlated with plasma viral loads (p<0.0001, r = 0.90, Fig 3A). We calculated the absolute numbers of p24+ cells per μL of blood and determined linear regression between plasma viral load (copies/μL) and the absolute number of p24+ cells per μL of blood (p<0.0001; r = 0.95, log(Y) = 0.67*log(X)–2.4; Fig 3B). Based on this analysis, we estimated that each individual p24+ cell detected in the blood was associated to approximately 2,000 viral particles in the plasma.

**Table 1:** Characteristics of viremic individuals.

ID	Age (years)	Gender^1^	CD4 count (cells/μl)	CD4/CD8 Ratio	Viral Load (copies/mL)	Time since diagnosis (years)
**VIR1**	22	M	750	0.8	286	1.0
**VIR2**	46	M	504	0.7	667	7.3
**VIR3**	37	F	415	0.5	1256	5.0
**VIR4**	55	M	480	0.3	3300	5.7
**VIR5**	32	M	543	0.6	6077	N.A
**VIR6**	41	M	406	0.4	6671	22.6
**VIR7**	42	F	638	1.3	8700	7.6
**VIR8**	38	M	138	0.4	14614	11.0
**VIR9**	43	F	681	1.3	15000	8.2
**VIR10**	47	F	779	0.6	18320	13.5
**VIR11**	29	F	284	0.5	21000	5.3
**VIR12**	47	M	492	1.0	39489	8.5
**VIR13**	49	M	281	0.4	44848	7.3
**VIR14**	27	F	319	0.7	51000	12.0
**VIR15**	40	M	434	0.2	51000	2.5
**VIR16**	41	M	499	0.3	52915	N.A
**VIR17**	45	M	228	0.3	73109	10.3
**VIR18**	38	M	320	0.2	132886	0.4
**VIR19**	43	M	356	0.3	193437	1.9
**VIR20**	51	M	289	0.1	268432	13.1
VIR21*	34	M	688	0.2	366487	0.2
*Median*	*41*		*434*	*0*.*4*	*21000*	*7*.*3*
*IQ range*	*[37–46]*		*[319–543]*	*[0*.*3–0*.*7]*	*[6671–52915]*	*[3*.*8–10*.*6]*

^1^Gender: F = female, M = male

N.A = not available

* acute stage of HIV infection and excluded from the analysis in Fig 3A

To evaluate the ability of HIV-Flow at estimating the size of the persistent HIV reservoir in ART-suppressed individuals, we used samples from a cohort of 24 well-characterized participants on ART for a median of 9.9 years (Table 2, ART1-24). Upon stimulation of CD4+ T cells with PMA/ionomycin, we measured a median frequency of 4.3 p24+ cells/10^6^ cells (IQR 0.7–8.0, Table 2). This frequency of infected cells was intermediate between mQVOA (median = 0.9 IUPM) and TILDA (median = 20.1 cells/10^6^ CD4+ T cells) and dramatically lower than the frequency of cells harboring HIV DNA (medians of 1,014 and 510 copies/10^6^ cells for total and integrated HIV DNA, respectively, Fig 3C). Interestingly, the frequency of p24+ cells measured by HIV-Flow correlated with the levels of total HIV DNA (r = 0.56, p = 0.004), integrated HIV DNA (r = 0.57, p = 0.004) and with the frequency of cells producing Tat/rev RNA measured by TILDA (r = 0.65, p = 0.0005, Fig 3D). Although the association was weaker, we also observed a statistically significant correlation between the frequencies of infected cells measured by HIV-Flow and mQVOA (r = 0.43, p = 0.04, Fig 3D). Taken together, our results indicate that the HIV-Flow assay, which detects cells harboring translation-competent genomes, measures frequencies of infected cells that are intermediate between viral RNA induction assays (transcription-competent genomes) and mQVOA (replication-competent genomes). In addition, our results suggest that this novel assay may be used as a surrogate for other methods aimed at measuring the size of the HIV reservoir in virally suppressed individuals.

**Table 2:** Characteristics of ART-suppressed individuals.

ID	Age (years)	Gender^1^	CD4 count (cells/μl)	CD4/CD8 Ratio	Viral Load (copies/mL)	Time to ART initiation (years)	Time since diagnosis (years)	Time on ART (years)
**ART1**	35	M	1589	1.0	˂ 40	0.8	8.0	7.1
**ART2**	50	M	331	0.7	˂ 40	1.7	20.0	18.3
**ART3**	55	M	696	0.7	˂ 40	6.0	15.4	9.4
**ART4**	52	M	836	0.5	˂ 40	0.4	14.0	14.0
**ART5**	45	M	429	0.8	˂ 40	10.7	14.0	3.3
**ART6**	36	M	461	0.5	˂ 40	0.5	7.0	6.6
**ART7**	60	M	624	1.1	˂ 40	0.4	4.6	4.3
**ART8**	67	M	620	1.1	˂ 40	2.1	20.4	18.3
**ART9**	48	M	662	1.0	˂ 40	0.2	16.9	16.7
**ART10**	36	M	882	0.7	˂ 40	4.7	13.8	9.1
**ART11**	55	M	847	1.4	˂ 40	15.1	29.3	14.1
**ART12**	21	M	331	0.8	˂ 40	0.2	4.3	4.1
**ART13**	66	M	601	0.7	˂ 40	6.5	22.2	15.6
**ART14**	60	M	803	1.9	˂ 40	4.8	25.1	20.4
**ART15**	58	M	628	1.0	˂ 40	0.9	21.6	20.7
**ART16**	58	M	520	0.4	˂ 40	14.0	21.4	7.4
**ART17**	56	M	582	1.4	˂ 40	1.4	11.7	10.3
**ART18**	47	M	625	1.2	˂ 40	0.3	15.1	14.8
**ART19**	36	M	471	1.5	˂ 40	7.8	12.0	4.2
**ART20**	45	M	681	0.6	˂ 40	1.1	13.4	12.3
**ART21**	58	M	602	2.1	35.0	0.8	9.2	8.4
**ART22**	57	M	1173	1.0	< 20	0.6	20.7	20.1
**ART23**	33	M	1042	1.3	˂ 40	3.0	10.8	7.8
**ART24**	44	M	914	0.7	˂ 40	1.0	5.9	4.9
**ART25**	41	M	1177	1.3	< 50	N.A	9.3	N.A
**ART26**	60	M	677	0.9	< 20	N.A	N.A	N.A
**ART27**	46	M	887	0.8	< 20	2.0	18.3	16.3
**ART28**	46	F	527	0.3	< 20	1.0	7.4	6.4
**ART29**	43	F	351	1.0	< 41	11.7	15.4	3.7
**ART30**	32	M	734	1.0	< 20	4.9	7.4	2.5
**ART31**	50	M	1110	2.8	< 20	N.A	16.0	N.A
**ART32**	46	F	1030	0.9	< 20	N.A	18.6	N.A
**ART33**	56	M	715	1.0	140.0	N.A	27.6	N.A
**ART34**	46	M	1897	2.3	< 20	N.A	19.7	N.A
**ART35**	32	M	248	0.7	< 20	0.5	3.1	2.7
**ART36**	31	M	394	0.5	49.0	2.9	3.7	0.9
**ART37**	63	M	782	0.5	< 40	13.0	19.3	6.3
**ART38**	47	M	356	0.4	< 40	10.3	14.7	4.4
**ART39**	39	M	616	0.5	< 40	0.6	1.8	1.2
*Median*	*47*		*662*	*0*.*9*		*1*.*7*	*14*.*4*	*7*.*8*
*IQ range*	*[40–57]*		*[524–865]*	*[0*.*7–1*.*2]*		*[0*.*6–6*.*0]*	*[8*.*3–19*.*6]*	*[4*.*3–14*.*8]*

^1^Gender: F = female, M = male

N.A = not available

### Phenotypic analysis of infected cells in untreated HIV infection

To determine if specific cell subsets are enriched in productively infected cells during untreated HIV infection, we developed several panels of antibodies (S2 Table) to simultaneously quantify and phenotype p24+ cells in samples from viremic individuals. CD4+ T cells from 8 untreated participants were rested for 18h and stained for cell-surface markers and intracellular p24.

**Fig 4:**
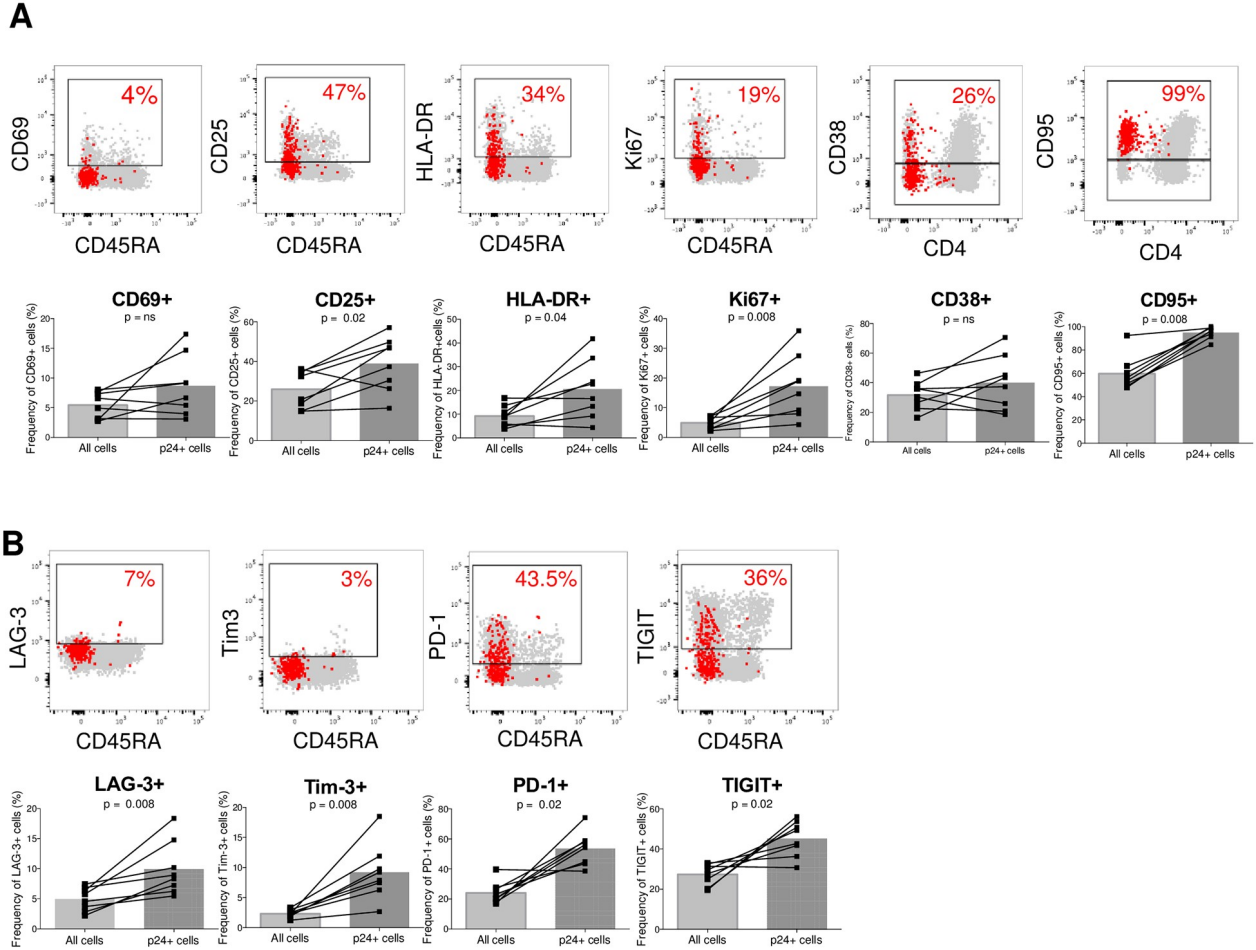
p24-producing cells from untreated individuals are enriched in subsets expressing markers of activation and exhaustion. Representative dot plots showing the phenotype of p24+ cells (represented as red dots) overlaid onto all cells (in grey) after 18h of resting. The contribution of a given subset to the pool of all cells and to the pool of infected cells (p24+) are compared in samples from n = 8 untreated individuals (VIR9, 10, 12, 13, 15, 16, 20, 21). (A) Activation/proliferation/apoptosis markers: CD69, CD25, HLA-DR, Ki67, CD38, CD95. (B) Immune checkpoint molecules: LAG-3, Tim-3, PD-1, TIGIT. For statistical analyses, Wilcoxon matched-pairs signed rank test was performed.

Since T cell activation is required for productive infection [51], we first assessed whether CD4+ T cells displaying an activated phenotype were enriched in productive HIV. Several markers of T cell activation or proliferation were significantly enriched in productively infected cells, including CD25 (p = 0.02), HLA-DR (p = 0.04) and Ki67 (p = 0.008) (Fig 4A, S5A Fig, and S3 Table). In contrast, p24+ cells were not enriched in CD4+ T cells expressing CD69 and CD38, suggesting that a restricted number of activation markers are associated with productive HIV infection. Furthermore, the death receptor Fas (CD95), which expression increases upon T cell activation [52, 53], was expressed by 95% of p24+ cells (p = 0.008, Fig 4A, S5A Fig and S3 Table). To determine if the combination of multiple markers of activation/proliferation could further enrich in p24+ cells, we performed a Boolean analysis with the receptors we identified as preferentially expressed by p24+ cells (CD25, HLA-DR, CD95, Ki67). In the majority of the samples tested, the frequency of p24+ cells progressively increased with the number of markers co-expressed (median frequencies = 3, 90, 174, 387, and 745, for cell subsets expressing 0, 1, 2, 3, 4 markers, respectively, S6A Fig).

Since T cell activation leads to the upregulation of several immune checkpoint molecules [54], we also measured the expression of LAG-3, Tim-3, PD-1 and TIGIT in p24+ cells. Strikingly, all these immune checkpoint molecules significantly enriched in productively HIV-infected cells (LAG-3, p = 0.008; Tim-3, p = 0.008; PD-1, p = 0.02 and TIGIT, p = 0.02; Fig 4B, S5A Fig and S3 Table). By performing a Boolean analysis with these 4 markers, we observed that the highest frequencies of p24+ cells were detected in cell subsets co-expressing 3 or 4 immune checkpoint molecules (median frequencies = 72, 134, 131, 539, and 403, for cell subsets expressing 0, 1, 2, 3, 4 molecules, respectively S6A Fig).

**Fig 5:**
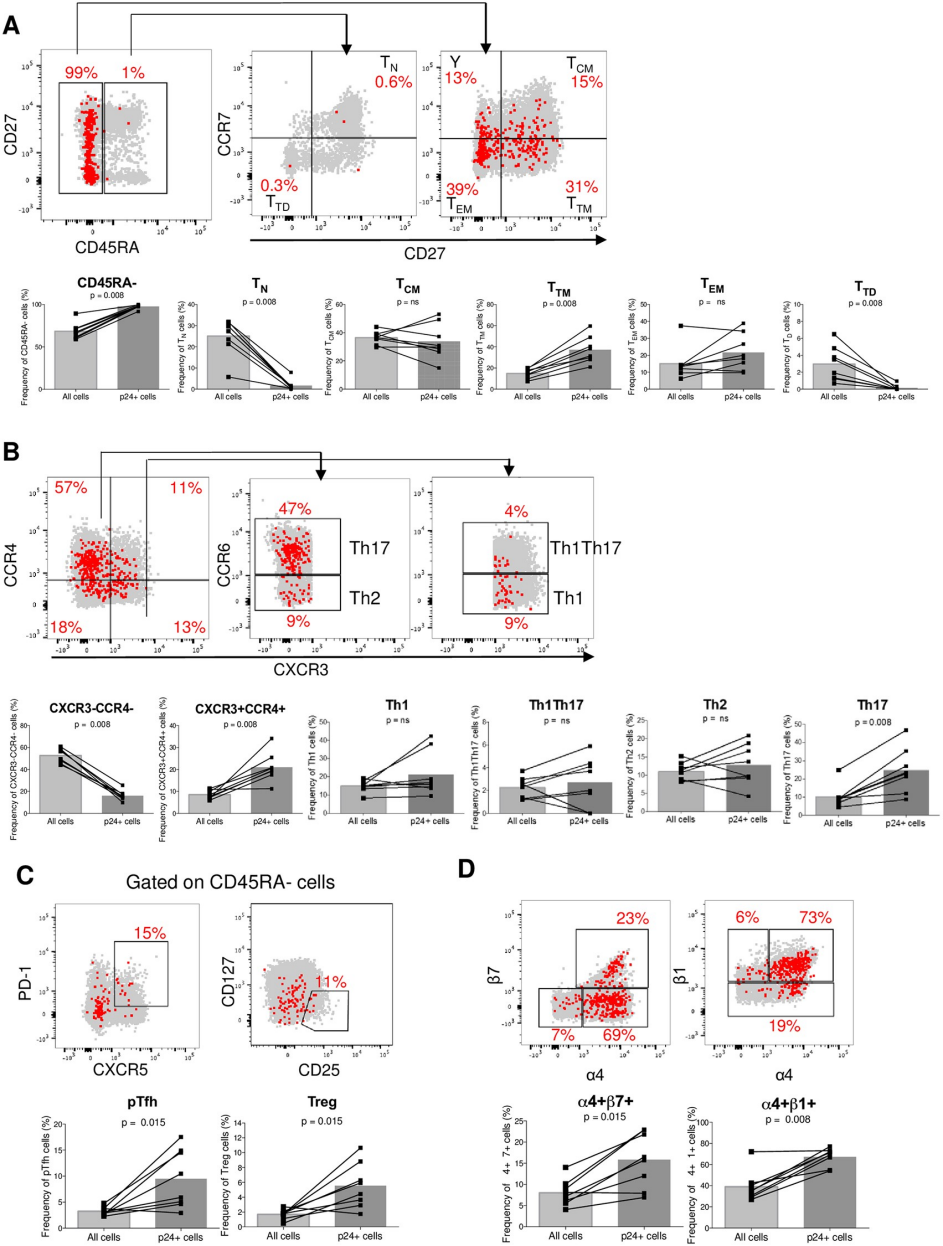
p24-producing cells from untreated individuals are enriched in various cell subsets. Representative dot plots showing the phenotype of p24+ cells (represented as red dots) overlaid onto all cells (in grey) after an 18h-resting. The contribution of a given subset to the pool of all cells and to the pool of infected cells (p24+) are compared in samples from n = 8 untreated individuals (same as in Fig 4). (A) Memory phenotype: T_N_, T_CM_, T_TM_, T_EM_ and T_TD_ cells. (B) CXCR3-CCR4-, CXCR3+CCR4+, Th1, Th2, Th17, and Th1Th17 cells. (C) pTfh and Treg cells. (D) Integrins: α4β7 and α4β1. For statistical analyses, Wilcoxon matched-pairs signed rank test was performed.

Since memory CD4+ T cells have previously been shown to be preferentially targeted by HIV [55], we then analyzed the memory status of p24+ cells by focusing on naïve (T_N_: CD45RA+CCR7+CD27+), central memory (T_CM_: CD45RA-CCR7+CD27+), transitional memory (T_TM_: CD45RA-CCR7-CD27+), effector memory (T_EM_: CD45RA-CCR7-CD27-) and terminally differentiated (T_TD_: CD45RA+CCR7-CD27-) cells (Fig 5A, S5A and S7A Figs and S3 Table). Productively infected cells were enriched in the T_TM_ subset (p = 0.008, Fig 5A) which encompassed 39% of all p24+ cells (S7A Fig). Conversely, p24+ cells rarely displayed a T_N_ or T_TD_ phenotype (1.9 and 0.2%, respectively). Although a significant fraction of p24+ cells also displayed a T_CM_ (36%) or T_EM_ (23%) phenotype, these subsets were not significantly enriched in infected cells during untreated HIV infection.

Several studies have shown that HIV preferentially infects CD4+ T cells endowed with specific effector functions, including Th17 [26, 27], T follicular helper cells (Tfh) [24, 46] and regulatory T cells (Treg) [56, 57]. We used HIV-Flow to assess the relative contribution of these subsets to the pool of productively infected cells. Using antibodies specific for CCR4, CXCR3 and CCR6, we identified 6 populations of cells as described previously [26]. The greatest levels of enrichment were observed in Th17 cells (CXCR3-CCR4+CCR6+) and CXCR3+CCR4+ cells which encompassed 25% (p = 0.008) and 21% (p = 0.008) of all p24+ cells, respectively (Fig 5B, S5A and S7A Figs and S3 Table). Conversely, p24+ were underrepresented within the CXCR3-CCR4- subsets (p = 0.008). Of note, some p24+ cells were detected within the Th1 (CXCR3+CCR4-CCR6-), Th1Th17 (CXCR3+CCR4-CCR6+) and Th2 subsets (CXCR3-CCR4+CCR6-) (21%, 2.8% and 13%, respectively), but none of these subsets were significantly enriched in infected cells (Fig 5B, S5A and S7A Figs and S3 Table). In contrast, both peripheral T follicular helper cells (CXCR5+PD-1+) and regulatory T cells (CD25+CD127-) were enriched for p24+ cells (p = 0.015 for both subsets) and encompassed 9.5% and 5.5% of infected cells, respectively (Fig 5C and S3 Table).

The integrin α4β7 defines a T cell subset that is highly susceptible to HIV infection [58, 59]. Using HIV-Flow, we observed that cells expressing this homing receptor were enriched in p24+ cells (p = 0.015, respectively, Fig 5D, S5A Fig and S3 Table). Interestingly, we also observed a significant enrichment in p24+ cells within α4+β1+ cells (p = 0.008). Taken together, our results indicate that productively infected cells are enriched in specifically differentiated (T_TM_) and effector (Th17, CXCR3+CXCR4+, Tfh, Treg cells) subsets as well as in cells expressing immune activation markers, immune checkpoint molecules and homing receptors.

### Phenotypic analysis of persistently infected cells in virally suppressed individuals

To identify the phenotype of persistently infected cells during ART, we stimulated CD4+ T cells from 12 virally suppressed individuals with PMA/ionomycin and used several panels of antibodies as above (S2 Table). Since PMA/ionomycin led to increased expression of several cell surface markers of interest, we stimulated CD4+ T cells in the presence of brefeldin A (BFA) to prevent upregulation of these molecules. Although most of the markers of interest retained their expression using this approach (S8 Fig), analysis of functional subsets was not possible due to dramatic changes in the expression of CCR6, CXCR3, CCR4, CXCR5, CD25 and CD127, even in the presence of BFA (S9A and S9B Fig). In addition, CD4 downmodulation was still observed after PMA/ionomycin stimulation despite the use of BFA, which did not allow us to assess CD4 expression in p24+ cells from ART-suppressed individuals (S9C Fig).

**Fig 6:**
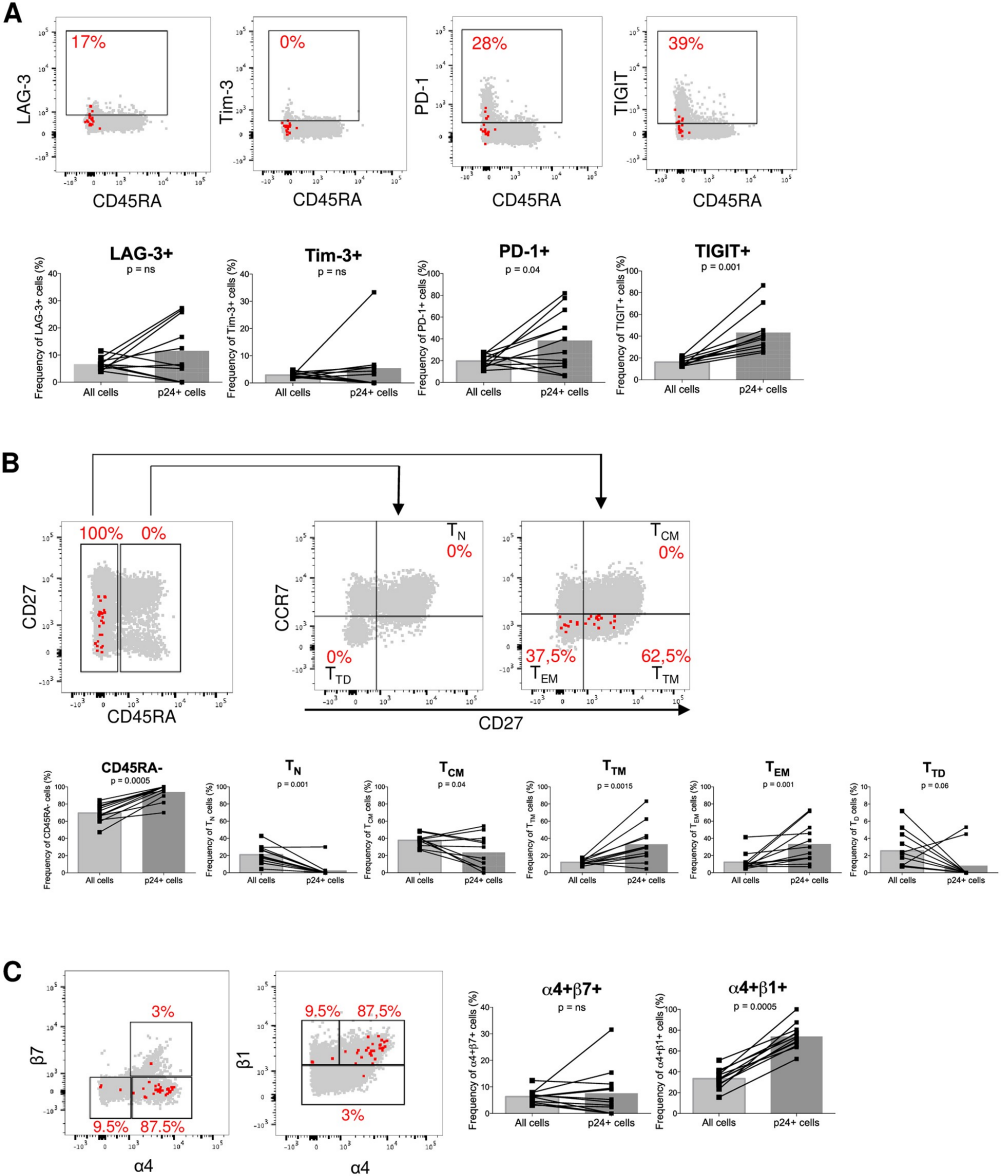
p24-producing cells from ART-suppressed individuals are enriched in T_TM_/T_EM_, PD-1+, TIGIT+ and α4+β1+ cells. Representative dot plots showing the phenotype of p24+ cells represented as red dots overlaid onto all cells in grey. Purified CD4 T cells from 12 ART-suppressed individuals were stimulated with PMA/ionomycin for 24h before analysis. BFA was added 1h before PMA/ionomycin stimulation and was maintained until the end of the stimulation. The contribution of a given subset to the pool of all cells and to the pool of infected cells (p24+) are compared in samples from n = 12 ART-suppressed individuals (ART5, 9, 10, 11, 14, 16, 18, 19, 21, 22, 27, 33). (A) Immune checkpoint molecules: LAG-3, Tim-3, PD-1, TIGIT. (B) Memory phenotype: T_N_, T_CM_, T_TM_, T_EM_ and T_TD_ cells. (C) Integrins: α4β7 and α4β1. For statistical analyses, Wilcoxon matched-pairs signed rank test was performed.

We previously demonstrated that the immune checkpoint molecules PD-1, LAG-3 and TIGIT are preferentially expressed at the surface of CD4+ T cells harboring integrated HIV DNA [21]. We used HIV-Flow to determine if these cell surface markers would also enrich in translation-competent genomes. PD-1 and TIGIT expressing cells were enriched in p24-producing cells (p = 0.04 and p = 0.001, respectively, Fig 6A, S5B Fig and S3 Table), whereas p24+ cells were not preferentially detected in CD4+ T cells expressing LAG-3 and Tim-3. We performed a Boolean analysis to determine if the combination of PD-1 and TIGIT further enriched in p24+ cells. Interestingly, p24+ cells were rarely found in cells expressing none of these markers, whereas cells expressing one or two markers were significantly enriched in p24+ cells compared to all cells. Of note, in 5/11 individuals, the degree of enrichment was further increased when PD-1 and TIGIT were co-expressed compared to cells expressing only one marker (S6B Fig).

Several studies have demonstrated that HIV genomes preferentially persist in CD4+ T cells displaying a memory phenotype, including T_CM_, T_TM_ and T_EM_ cells [60–62]. Analysis of the memory phenotype of p24+ cells by HIV-Flow revealed that the majority of cells carrying translation-competent genomes displayed a T_TM_ or T_EM_ phenotype (35.4% and 35.7%, p = 0.0015 and p = 0.001, respectively), whereas p24+ T_N_ and T_TD_ were rare in individuals on ART (2.9% and 0.9%, respectively, Fig 6B, S5B and S7B Figs and S3 Table). Although a significant fraction of p24+ cells displayed a T_CM_ phenotype (25.2%), infected cells were slightly underrepresented within this subset (p = 0.04). Unlike what we observed in samples from untreated individuals, the α4+β7+ subset was not enriched in p24+ cells in virally suppressed individuals (Fig 6C, S5B Fig and S3 Table). In contrast, the α4+β1+ population remained significantly enriched in p24-producing cells during ART (p = 0.0005), with 74% of p24+ cells expressing this homing receptor on their surface.

Since CD32a was recently proposed as a marker of CD4+ T-cell harboring replication-competent proviruses during ART [12], we used HIV-Flow to determine if p24-producing cells from ART-suppressed individuals were enriched in the CD32^high^ population. Following stimulation with PMA/ionomycin in the presence of BFA, we observed a slight increase in the proportion of cells expressing CD32a (0.15% to 0.32% CD32+ cells, S10A Fig). In samples from four ART-suppressed individuals, none of the p24-producing cells were found to express high levels of CD32 (S10B Fig), indicating that the majority of the cells harboring translation competent HIV do not express CD32 in individuals on ART.

Altogether, our results indicate that the cells in which HIV persists during ART are phenotypically diverse, with significant enrichment for translation-competent HIV genomes in cells displaying differentiated memory phenotypes (T_TM_ and T_EM_ cells) as well as in subsets of cells expressing PD-1, TIGIT and α4β1.

To determine if the cell surface markers we identified as preferentially expressed by infected cells could be used in combination to further enrich in p24+ cells, we combined antibodies to CD45RA, PD-1, TIGIT and α4β1 in a single antibody panel. We observed that the highest degree of enrichment was obtained in cells displaying a CD45RA-, α4β1+, TIGIT+ phenotype (5.4-fold enrichment, p = 0.008, S11 Fig).

## Discussion

An increasing number of interventions aimed at reducing the size of the HIV reservoir are currently under evaluation in clinical trials [11]. Measuring the efficacy of these strategies requires precise quantification of the frequency of infected cells [29, 63]. The phenotypic characterization of these long-lived reservoir cells is also of great interest since several eradication strategies target specific cellular reservoirs. Currently, mRNA Flow-FISH, which relies on the simultaneous detection of HIV transcripts and p24 protein by flow cytometry [46], is the only approach that can simultaneously measure the frequency and assess the phenotype of infected CD4+ T cells persisting in ART-suppressed individuals. Herein, we developed a simpler version of the mRNA flow FISH assay that requires only 5–10 million CD4+ T cells, can be completed in two days, and can be used on a large scale to quantify and phenotype the translation-competent HIV reservoir. We found that HIV-Flow is reproducible and linear to the lowest dilution tested. Cell sorting of p24- and p24+ cells revealed that p24+ cells harbored on average 1 to 1.5 HIV DNA copies per cell, whereas 0 to 0.02 HIV genomes/cell were measured in p24- cells. Of note, we measured 1.0–1.5 copies of HIV DNA per p24+ cell (average = 1.25 copies/cell), which is slightly higher than the frequency of 1 copy per cell previously reported [64]. This may be explained by multiple infections of a single cell or by the relative imprecision of the quantitative PCR assay. In addition, although the majority of single sorted p24+ cells harbored detectable HIV DNA, HIV genome could not be amplified in 25–40% of p24+ single cells, likely reflecting the inefficient amplification of a single viral genome.

Using HIV-Flow, we measured a median frequency of 4.3 p24-producing cells/10^6^ cells in ART-suppressed individuals. This is 200 times lower than the frequency of cells harboring total HIV DNA (1,014 cells/10^6^ cells), reflecting an important gap between the total number of HIV DNA molecules and the small fraction of genomes that can lead to the production of a correctly folded p24 protein. This is in line with the high proportion of genomes carrying hypermutations or internal deletions as previously reported [30, 40, 65]. Similarly, HIV-Flow frequencies are 4.7 times lower than those measured by TILDA (median = 20.1 copies/10^6^ CD4 T cells), suggesting that a significant proportion of proviruses may give rise to msRNA upon stimulation but not to p24 protein. The frequency of p24-producing cells measured by HIV-Flow was also 4.8 times higher than the one measured by mQVOA (median = 0.9 IUPM), suggesting that a fraction of p24-producing cells do not harbor replication-competent proviruses likely as a consequence of defects in other viral genes. This difference in frequencies between HIV-Flow and mQVOA may also be explained if spreading infection is not fully efficient following the induction of replication competent proviruses in co-culture. In spite of these dramatic differences in these measures, we observed that the frequencies of p24-producing cells assessed by HIV-Flow correlated with several other assays aimed at quantifying the size of the reservoir (integrated HIV DNA, TILDA, and mQVOA). Taken together, our results indicate that, as expected, HIV-Flow measures a frequency intermediate between viral RNA induction assay and replication-competent HIV and may be used as a surrogate for several of these assays.

We took advantage of this approach to identify cellular subsets enriched in HIV-infected cells during untreated and treated infection. Strikingly, the vast majority (97.6%) of productively infected T cells displayed low levels of CD4 on their surface, likely as a result of Nef, Vpu and/or Env-mediated downregulation, as previously proposed [48–50]. This indicates that the functions of Nef/Vpu/Env are preserved in the majority of p24-producing cells, suggesting that Gag production is usually accompanied by the production of other functional HIV proteins. Productively infected cells were characterized by higher levels of immune activation as demonstrated by heightened expression levels of several activation markers including CD25, HLA-DR and Ki67. This is in line with previous studies reporting that proliferating activated CD4+ T lymphocytes are more susceptible to HIV infection compared to resting CD4+ T cells [51, 66]. Accordingly, several immune checkpoint molecules which are upregulated during T cell activation like PD-1, TIGIT, LAG-3, Tim-3 [54] were also preferentially expressed by productively infected cells. We also assessed the memory phenotype of the infected cells and observed that productively infected CD4+ T cells were more likely to display a transitional memory phenotype than their uninfected counterparts, as previously reported in *in vitro* infection studies [67]. Conversely, p24+ cells were rarely detected within the naive and terminally differentiated subsets, which may be explained by the relative resistance of naive CD4+ T cells to HIV-infection by CCR5-using strains [68, 69] and by the short life-span of terminally differentiated cells. Using chemokine receptors to predict the functionality of these cells, we observed that the Th17 subsets encompassed the largest proportion of productively infected CD4+ T cells (25%). This is in accordance with previous studies in which CCR6+ T cells were shown to be highly permissive to HIV infection [27], likely as a result of their heightened expression of HIV dependency factors [70]. We also observed that peripheral Tfh cells and Treg cells were enriched in productively infected cells, which is also supported by previous findings [24, 46, 56, 57]. In fact, peripheral Tfh represent the circulating counterparts of germinal center Tfh cells [71], which have been shown to serve as the major compartment for HIV production and replication during untreated HIV infection [24].

In the SIVmac251 model, peripheral memory CD4 T cells expressing high levels of α4β7 were shown to be preferentially infected during the very early phase of infection [72] and to be preferentially depleted from gut tissues as early as the first 2 weeks following infection in humans [59]. In line with these data, we observed that p24-producing cells were enriched in the α4+β7+ subset in 7/8 untreated individuals, which is consistent with their ability to home to the gut-associated lymphoid tissue (GALT), a compartment in which inflammation is heightened during chronic untreated HIV infection and in which HIV replication may be favoured. Interestingly, we identified the integrin α4β1 as a cell surface molecule expressed by a large fraction of productively infected cells. Since α4β1 drives homing to the inflamed central nervous system [73] and to the bone marrow [74], our results suggest that these compartments may also contribute to HIV replication during untreated HIV infection.

Altogether, our results demonstrate the broad diversity of the cells in which HIV replicates during untreated HIV infection including cells displaying an activated phenotype, cells expressing immune checkpoint molecules, transitional memory cells, Th17, Tregs, pTfh as well as T cells expressing homing receptors to inflamed tissues.

The identification of a cellular marker specifically expressed by persistently infected cells in individuals on ART remains elusive. Although the Fc-gamma receptor CD32 was originally proposed to mark these cells [12], our own measures using HIV-Flow as well as other recent studies did not confirm these observations [13–18]. Rather, our results suggest that the latent reservoir, similar to the pool of productively infected cells in untreated individuals, is broadly diverse. Yet, we identified T cell phenotypes that are enriched in p24-producing cells in the blood of individuals on ART. There are multiple reasons why specific cellular proteins would be preferentially expressed at the surface of p24-producing cells: these molecules could be upregulated as a consequence of HIV infection or could be preferentially expressed by cells highly sensitive to HIV infection. Alternatively, infected cells expressing these markers may have a selective advantage to persist over time.

Although infected cells were detected in all memory subsets as reported previously [60], we found T_TM_ and T_EM_ cells to be particularly enriched in p24+ cells, which is consistent with the fact that these subsets contain the largest fraction of intact proviruses [40]. We also observed that CD4+ T cells expressing PD-1 and TIGIT are enriched in infected cells, as we previously reported using quantification of integrated HIV DNA and TILDA [21]. Unlike what we observed in untreated HIV infection, we did not observe an enrichment of p24-producing cells in the α4+β7+ population in virally suppressed individuals, possibly as a result of the retention of these cells in the GALT. In contrast, enrichment in α4+β1+ cells persisted after viral suppression. This may result from a combination of functions ensured by α4β1. Indeed, α4β1 not only mediates the adhesion and transendothelial migration of leukocytes, but also provides costimulatory signals that contribute to the activation of T lymphocytes [75], which may facilitate infection of T cells expressing α4β1 through proximate contact. In addition, α4+β1+ cells not only home to the inflamed CNS, but also preferentially migrate to secondary lymphoid organs [76, 77], which represent important HIV reservoirs during ART. Therefore, α4β1 expression may facilitate HIV persistence by enhancing T cell expansion, a major mechanism of HIV persistence during ART, as well as through migratory effects.

We acknowledge several limitations to our study. First, HIV-Flow may underestimate the frequency of p24-producing cells in samples from viremic individuals due to high levels of cell death and cellular turnover when compared to cells from ART-suppressed individuals [78]. Moreover, it is likely that a fraction of the cells harboring translation-competent viruses would require more than one round of stimulation to be reactivated, as it was described for QVOA [30]. Unlike QVOA, HIV-Flow does not measure replication-competent HIV and it is likely that a significant fraction of p24+ cells measured in our assay does not produce intact viral particles that have the ability to replicate in culture. Since Rev and RRE are not essential for Gag expression, proviruses with massive 3’ deletions could still produce p24 [79, 80]. Therefore, obtaining full length HIV sequences in p24+ cells will be important to determine the fraction of translation competent genomes that are intact. This overestimation of the size of the reservoir may vary between individuals and whether the frequency of infected cells measured by HIV-Flow can be used to predict control of HIV replication during analytic treatment interruption remains to be determined. Another limitation of HIV-Flow is the need to activate CD4+ T cells to detect intracellular p24 in samples from virally suppressed individuals, which results in alteration in the phenotype of the cells. Although this phenomenon can be attenuated by adding BFA during the stimulation, several cell surface markers, including CD4, could not be analyzed using our assay. Future studies will also need to investigate the possibility of detecting and characterizing p24+ cells in tissues, since anatomical compartments carry the majority of persistent HIV/SIV during ART [81, 82]. Although the HIV-Flow assay only requires relatively limited numbers of cells, we agree that obtaining 5–10 million CD4+ T cells may be challenging in clinical studies in which repeated samplings are required or in pediatric studies. In addition, the limited number of cells that can be recovered from tissue sampling may not allow sufficient sensitivity to detect p24+ cells by HIV-Flow. Future studies will also be needed to determine whether various HIV clades can be recognized by the two antibodies used to detect p24.

In conclusion, we developed a new flow cytometry-based assay that allows the simultaneous quantification and phenotyping of p24-producing cells both in untreated and ART-suppressed individuals. By combining our assay and novel flow cytometry or mass cytometry approaches that allow the analysis of 30–50 parameters simultaneously, the identification of cellular subsets highly enriched in HIV in blood and tissues from individuals living with HIV could become a more realistic endeavor.

## Materials and methods

### Participants and blood collection

All participants underwent leukapheresis to collect large numbers of PBMCs. A total of 21 untreated viremic individuals and 39 individuals on stably suppressive ART participated in this study. Participants characteristics are summarized in Tables 1 and 2. PBMCs were isolated by Ficoll density gradient centrifugation and were cryopreserved in liquid nitrogen.

### Ethics statement

All participants were adults and signed informed consent forms approved by the McGill University Health Centre, the Centre Hospitalier de l’Université de Montréal and the Martin Memorial Health Systems review boards.

### Antibodies

We used a combination of two antibodies directed to p24: p24 KC57-PE was obtained from Beckman Coulter (6604667) and p24 28B7-APC was obtained from MediMabs (MM-0289-APC). The following antibodies were used for staining: CD3 (UCHT-1), CD4 (SK3), CD8 (RPA-T8), CD45RA (HI100), CCR7 (3D12), PD-1 (EH12.1), β1 (MAR4), β7 (FIB504), CD69 (FN50), CD25 (M-A251), HLA-DR (G46-6), CD38 (HIT2), Ki67 (MOPC-21), CD95 (DX2), CD39 (Tu66), CD127 (HIL-7R-M21), CXCR3 (1C6), CCR4 (1G1), CCR6 (11A9) were purchased from BD Bioscience. LAG-3 (FAB2319) was obtained from R&D systems, and TIGIT (MBSA43) from eBioscience. CD27 (O323), Tim-3 (F38-2E2), α4 (9F10), CD28 (CD28.2) and CD32 (FUN-2) were purchased from BioLegend. Live/Dead Aqua Cell Stain (405nm) was obtained from ThermoFisher Scientific (L34957). Detailed panels of antibodies are reported in S2 Table.

### HIV-flow procedure

Upon thawing of PBMCs, CD4+ T cells were isolated by negative magnetic selection using the EasySep Human CD4 T Cell Enrichment Kit (StemCell Technology, 19052). Purity was typically >98%. 5-10x10^6^ CD4+ T cells were resuspended at 2x10^6^ cells/mL in RPMI + 10% Fetal Bovine Serum and antiretroviral drugs were added to the culture (200nM raltegravir, 200nM lamivudine). Cells were stimulated with 1μg/mL ionomycin (Sigma, I9657) and 25nM (18h) or 162nM PMA (24h) (Sigma, P8139) for samples from viremic and aviremic individuals, respectively. In phenotypic characterization experiments, samples from ART-suppressed individuals were pre-incubated for 1h with 5μg/mL Brefeldin A (BFA, Sigma, B2651) before stimulation in order to prevent the upregulation of cell surface markers. BFA was maintained in the culture until the end of the stimulation. After stimulation, cells were collected, resuspended in PBS and stained with the Aqua Live/Dead staining kit for 30min at 4°C. Cells were then stained with antibodies against cell surface molecules in PBS + 4% human serum (Atlanta Biologicals, 540110) for 30min at 4°C. After a 15min-fixation step at room temperature (RT) with 4% formaldehyde, cells were permeabilized for 30min at 4°C using the PermWash buffer (BD Biosciences, 554723), and stained with anti-p24 KC57 and anti-p24 28B7 antibodies for an additional 45min at RT. Cells were then washed and resuspended in PBS for subsequent analysis. In some experiments, the fixation/permeabilization step was performed with the FoxP3 Transcription Factor Staining Buffer Set (eBioscience, 00-5523-00) following the manufacturer’s instructions. Similar frequencies of p24+ cells were obtained using both permeabilization buffers (PermWash Buffer from BD versus FoxP3 Buffer Set from eBioscience; S12 Fig). Our detailed laboratory protocol describing all steps of the HIV-Flow procedure is accessible here: dx.doi.org/10.17504/protocols.io.w4efgte.

### HIV flow: Gating strategy and data analysis

The frequency of p24 double positive cells (KC57+, 28B7+) was determined by flow cytometry (BD LSRII) in gated viable CD4 T cells. This gate included both CD4 positive and CD4 negative T cells and was referred as “all cells” in this study. An example of the gating strategy is represented in S13 Fig. Samples with cell viability < 50% post-stimulation, as assessed by LIVE/DEAD staining, were excluded from the analysis. In all experiments, CD4+ T cells from an HIV-negative control were included to set the threshold of positivity. For gating strategy in immunophenotypic experiments, markers that are typically expressed at low levels in naïve cells (activation/proliferation markers, immune checkpoint molecules) were gated against CD45RA, whereas CD38 and CD95 were gated against CD4.

### Flow cytometry cell sorting

Cells were sorted in 96-wells PCR plates containing 15μL of proteinase K lysis buffer (0.1M Tris HCl, 0.5M KCl, 10mg/mL proteinase K from Life Technologies 25530–015). Single cells and up to a 1,000 cells per well were sorted on a BD FACS ARIA III. The PCR plates were subsequently incubated at 55°C for 16 hours for cell lysis followed by a 5min-incubation step at 95°C to inactivate proteinase K.

### Quantification of total and integrated HIV DNA

Frequencies of CD4+ T cells harboring total and integrated HIV DNA were measured by real time nested PCR, as previously described [83]. Briefly, cells were lysed by proteinase K digestion. Cell lysates were directly used for HIV DNA quantifications. Total HIV-1 DNA was amplified with primers annealing the LTR/*gag* region. Integrated HIV DNA was amplified with Alu primers together with a primer annealing the LTR. In all PCR reactions, primers specific for the CD3 gene were added to precisely quantify the cell input. In a second round of PCR, appropriate primers and probes were used to amplify HIV sequences from the first round of amplification. Inner primers specific for the CD3 gene were used in a separate reaction to determine cell input. The number of copies of total and integrated HIV-1 DNA were calculated by using serial dilutions lysed ACH-2 cells as a standard curve. All measures were performed in triplicate wells (except for plate cell sorting of p24+ cells). Results were expressed as numbers of HIV copies per million cells.

### Linearity of the assay

Purified CD4+ T cells from an HIV uninfected donor were activated with phytohemagglutinin-L (10 μg/ mL) for 48 hours and then maintained in RPMI 1640 complete medium supplemented with rIL-2 (100 U/mL). HIV NL4.3 was then used to infect activated primary CD4+ T cells by spin infection at 800 × g for 1 h in 96-well plates at 25 °C. *In vitro* infected CD4+ T cells were spiked at different ratios in CD4+ T cells purified from an HIV-uninfected control. The linearity of the assay was determined by comparing the predicted frequency of infected cells to the measured frequencies of p24+ cells obtained by single p24 staining with KC57 or 28B7 or by dual staining p24 KC57/p24 28B7.

### Tat/rev inducible limiting dilution assay (TILDA)

The frequency of CD4+ T cells with inducible multiply spliced HIV RNA was determined using TILDA as previously described [34].

### RNA-Flow FISH

The frequency of cells harboring simultaneously *gag* transcripts and Gag proteins was determined using RNA-Flow FISH, as previously described [46, 84].

### Modified quantitative viral outgrowth assay (mQVOA)

Purified CD4+ T cells were serially diluted in Costar plates coated with anti-CD3 (2.5μg/ml, Clone OKT3) and anti-CD28 (1μg/mL, Clone CD28.2, BioLegend 302902) monoclonal antibodies. Five serial 3-fold dilutions were performed at a starting concentration of 1x10^6^ cells/well (first dilution in a 24-well plate and following dilutions in a 96-well plate), with 6 replicates per dilution. After two days of stimulation, 50,000 or 10,000 MOLT-4/CCR5+ cells (NIH AIDS Reagent Program, 4984) were added to cell culture 24- or 96- well, respectively (day 0). Cell cultures were split twice weekly and half of cell culture supernatants (500μl or 100μl) were collected at days 7, 14 and 21 for quantification of soluble HIV-p24 protein. Supernatants were lysed and kept at -80°C until use. p24 protein was quantified by ELISA as previously described [85]. The number of wells positive for soluble p24 protein was determined, and the maximum likelihood method was applied to determine infectious units per million of cells (IUPM) (http://silicianolab.johnshopkins.edu/) [86].

### Statistical analyses

All data were analyzed using Graphpad Prism v6.0h. To compare frequencies of infected cells measured by total and integrated HIV DNA, TILDA, mQVOA and HIV-Flow, values were transformed in log_10_([HIV copies/10^6^ cells]+1). Results were represented as median or mean values, with interquartile range or minimum and maximum values, as indicated in the figure legends. Correlations were determined using nonparametric Spearman’s test. For enrichment data, non-parametric Wilcoxon matched-pairs signed rank tests were used. P values of less than 0.05 were considered statistically significant.

## Supporting information

S1 Fig Characterization of p24 KC57 and p24 28B7 antibodies. (A) Representative dot plots obtained from a binding competition experiment. Staining with either KC57 or 28B7 in a first step (1) does not prevent subsequent staining with the other antibody (2) (middle and right panels). Co-staining with both antibodies yielded a similar frequency of p24+ cells (left panel). (B) Representative dot plots showing single stainings or co-staining with p24 KC57 and p24 28B7 antibodies in samples from an HIV negative control and an ART-suppressed individual.
(TIF)

S2 Fig MFI of p24 antibodies following stimulation. Comparison of the MFI of the two p24 antibodies (p24 28B7-APC and p24 KC57-PE) in the presence or absence of stimulation with PMA/ionomycin in samples from 6 untreated individuals. The MFI of p24 antibodies was measured within the p24+ gate (p24 KC57+/p24 28B7+).
(TIF)

S3 Fig Single positive cells contain low HIV DNA levels. (A) Representative dot plot showing the gating strategy used to sort four populations of unstimulated cells (KC57+/28B7+, KC57+, 28B7+ and KC57-/28B7- cells) obtained from one untreated individual (VIR21). Total HIV DNA was quantified by ultrasensitive PCR in each sorted subset (right). (B) Levels of CD4 expression in the different subsets.
(TIF)

S4 Fig HIV DNA detection by PCR in p24+ single sorted cells. p24- and p24+ CD4 T cells from three ART-suppressed individuals were single sorted by flow cytometry and subjected to a duplex ultrasensitive PCR for the CD3 gene and the HIV genome (LTR/gag). Grey and dark circles represent successful detection of the CD3 gene and the HIV genome, respectively. A) 12 cycles of pre-PCR amplification were performed. B) 24 cycles of pre-PCR amplification were performed.
(TIF)

S5 Fig Frequencies of p24+ cells in different subsets. (A) Frequencies of p24+ cells in all cells and in each gated cellular subset in samples from 8 viremic individuals (same as in Figs 4 and 5). (B) Frequencies of p24+ cells in all cells and in each gated cellular subset in samples from 12 virally suppressed individuals (same as in Fig 6). Each sample is represented by a unique color-coded symbol. For statistical analyses, Wilcoxon matched-pairs signed rank test was performed: the median of each column was compared to the median of the first column (all cells). p*<0.05, p**<0.01, p***<0.001.
(TIF)

S6 Fig Boolean analysis. (A) Frequencies of p24+ cells in all cells and in cell subsets expressing 0, 1, 2, 3 or 4 markers in samples from 8 viremic individuals (same as in Figs 4 and 5). Analyses were performed on cells expressing CD25/CD95/HLA-DR/Ki-67 (top panel) and PD-1/TIGIT/LAG-3/Tim-3 (middle panel). (B) Frequencies of p24+ cells in all cells and in cell subsets expressing 0, 1 or 2 immune checkpoint molecules (PD-1/TIGIT) in samples from 11 virally suppressed individuals (same as in Fig 6). Each sample is represented by a unique color-coded symbol. For statistical analyses, Wilcoxon matched-pairs signed rank test was performed: the median of each column was compared to the median of the first column (all cells). p*<0.05, p**<0.01, p***<0.001.
(TIF)

S7 Fig Contribution of different subsets to the pool of p24+ cells. (A) Pie charts comparing the relative contributions of different subsets to the total pool of CD4 T cells (all cells, left) and to the pool of p24+ cells (right) in samples from viremic individuals. Contributions of memory subsets and effector subsets are represented. (B) Pie charts comparing the relative contributions of different subsets to the total pool of CD4 T cells (all cells, left) and to the pool of p24+ cells (right) in samples from ART-suppressed individuals. Contributions of memory subsets are represented.
(TIF)

S8 Fig Frequencies of CD4 T cell subsets before and after stimulation with PMA/ionomycin. (A) Representative dot plots showing the distribution of memory CD4 T cell subsets after 24h of resting or after 24h of stimulation with PMA/ionomycin + BFA in one representative ART-suppressed individual. (B) As in A) for LAG-3, Tim-3, PD-1 and TIGIT. (C) As in A) for α4β7 and α4β1.
(TIF)

S9 Fig Markers showing significant changes of expression following stimulation. (A) Representative dot plots showing the levels of expression of CXCR3/CCR4/CCR6 after 24h of resting or after 24h of stimulation with PMA/ionomycin + BFA in one representative ART-suppressed individual. (B) As in A) for CXCR5 and CD25. (C) As in A) for CD3 and CD4. Of note, the MFI of CD3 decreased after stimulation but the frequency of CD3+ cells remained unchanged.
(TIF)

S10 Fig p24+ cells from ART-suppressed individuals are not enriched in cells expressing high levels of CD32. Cryopreserved PBMCs from 4 ART-suppressed individuals were stimulated with PMA/ionomycin + BFA for 24h. (A) Representative dot plots of the CD32 staining in gated CD3+CD8- lymphocytes, CD3- lymphocytes and CD3-CD14+ monocytes, in the absence of stimulation (NS) and after PMA/ionomycin stimulation. (B) Dot plots showing CD32 expression in p24+ events (black) and in all cells (grey).
(TIF)

S11 Fig p24-producing cells from ART-suppressed individuals are highly enriched in CD45RA-α4β1+TIGIT+ cells. Frequencies of p24+ cells in all cells and in each gated cellular subset in samples from 8 ART-suppressed individuals. Each sample is represented by a unique color-coded symbol. Undetectable measurements are represented as open symbols. Mean folds-enrichment compared to all cells are indicated at the top of each bar. For statistical analyses, Wilcoxon matched-pairs signed rank test was performed: median values of each column were compared to the median of the first column (all cells). p*<0.05, p**<0.01.
(TIF)

S12 Fig Comparison of two permeabilization buffers for the detection of p24-producing cells by HIV-Flow. Dot plots showing the detection of p24+ cells in 2 samples, using 2 experimental conditions. Purified CD4 T cells from a viremic individual were rested for 18 hours, while purified CD4 T cells from an ART-suppressed individual were stimulated with PMA/ionomycin for 24h. The permeabilization step was performed either with the PermWash Buffer (BD) or with the FoxP3/Transcription Factor Staining Buffer Set (eBioscience).
(TIF)

S13 Fig Gating strategy used in HIV-Flow. Example of the gating strategy used for a representative sample following PMA/ionomycin stimulation of CD4+ T cells obtained from an ART-suppressed individual.
(TIF)

S1 Table Frequencies of infected cells measured by different assays in samples from ART-suppressed individuals. (DOCX)

S2 Table Panels of antibodies used for phenotyping of p24+ cells. (DOCX)

S3 Table Median fold differences in the frequencies of p24+ cells and all cells expressing a given cellular marker. (DOCX)
