# Supplementary figures and images for: Single-cell characterization and quantification of translation-competent viral reservoirs in treated and untreated HIV infection
Source: PLoS Pathog. 2019 Feb 27;15(2):e1007619. doi: 10.1371/journal.ppat.1007619 (PMC6411230; doi:10.1371/journal.ppat.1007619)

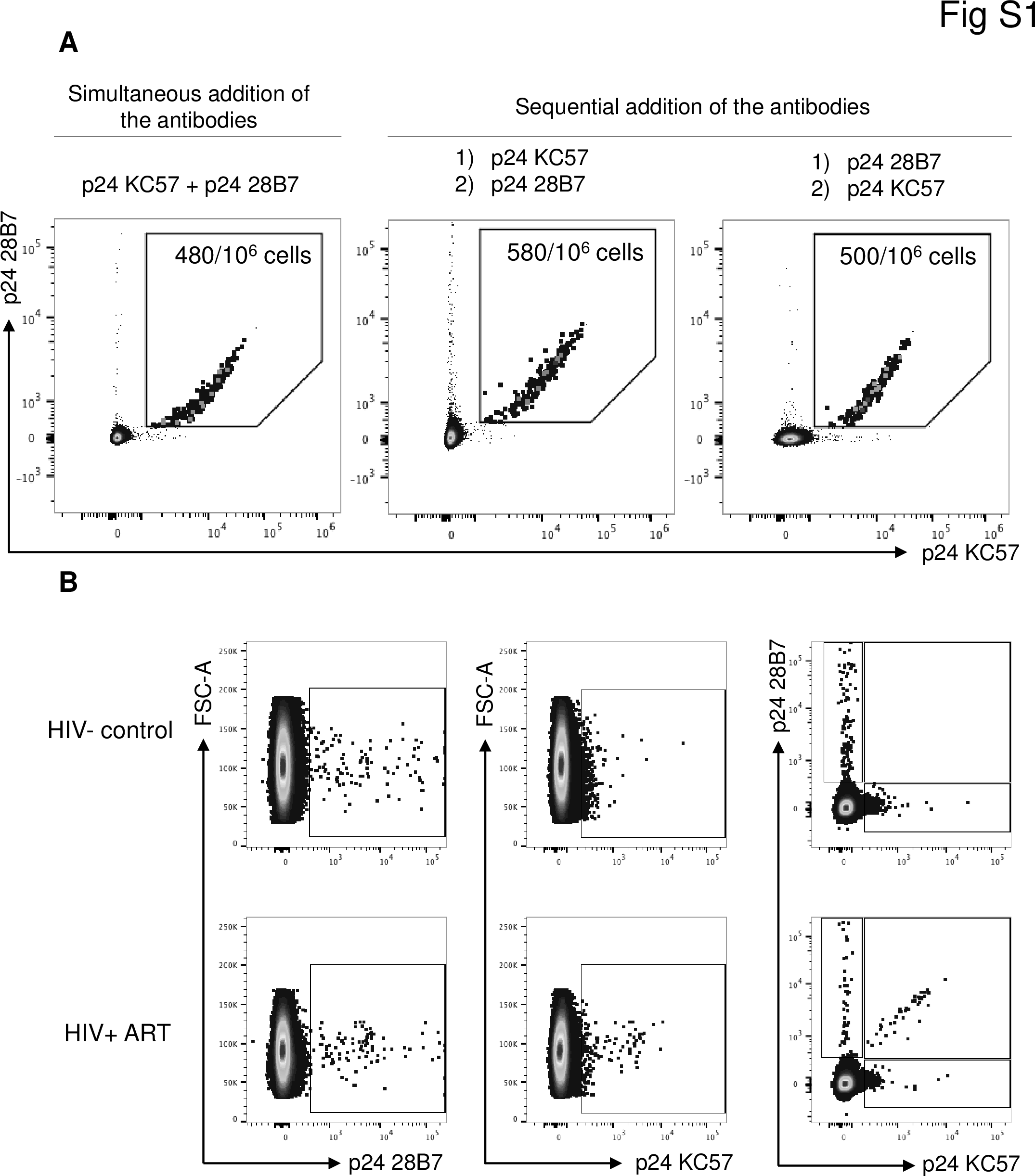

Supplement: S1 Fig — (A) Representative dot plots obtained from a binding competition experiment. Staining with either KC57 or 28B7 in a first step (1) does not prevent subsequent staining with the other antibody (2) (middle and right panels). Co-staining with both antibodies yielded a similar frequency of p24+ cells (left panel). (B) Representative dot plots showing single stainings or co-staining with p24 KC57 and p24 28B7 antibodies in samples from an HIV negative control and an ART-suppressed individual. (TIF) [file ppat.1007619.s001.tif]

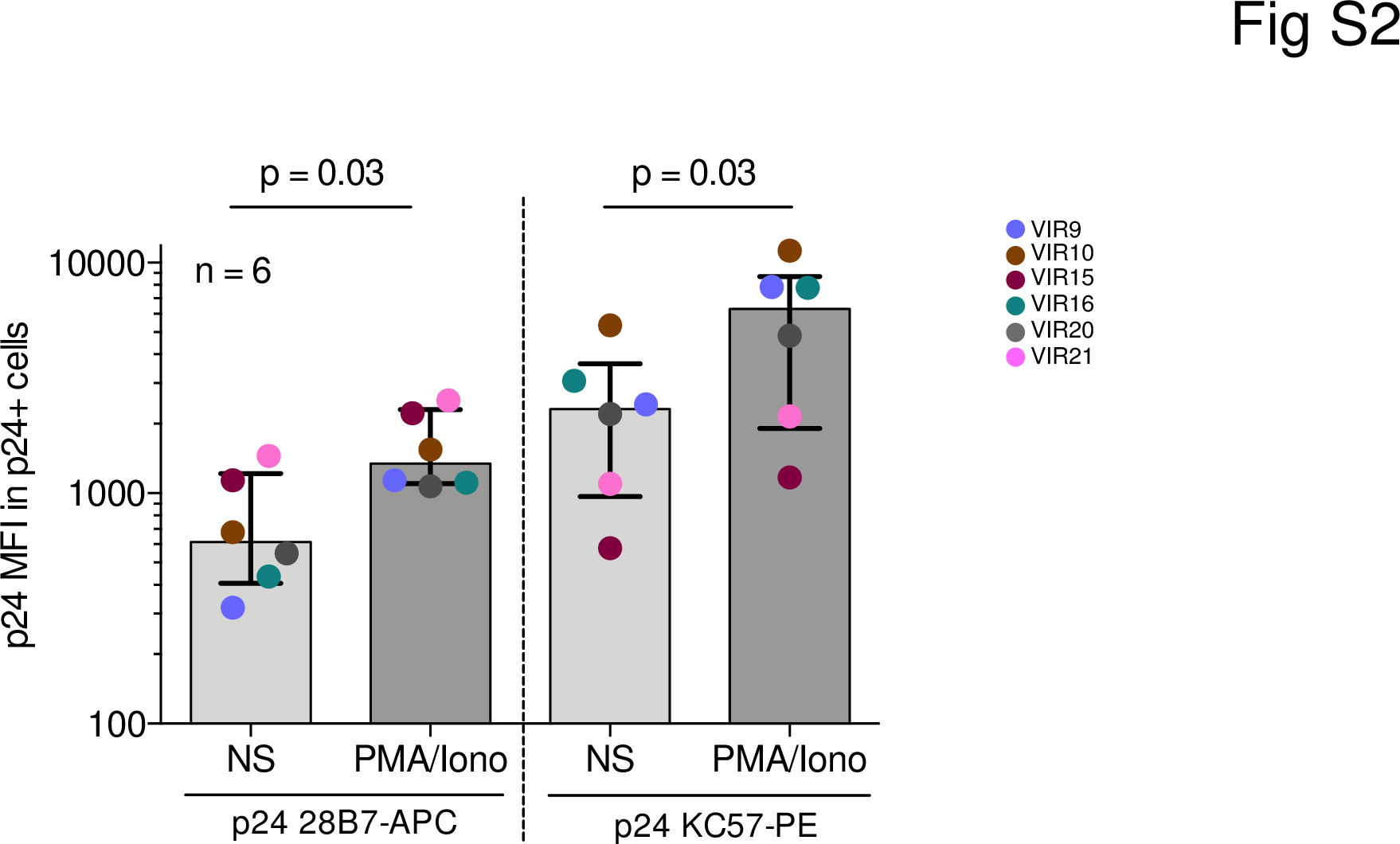

Supplement: S2 Fig — Comparison of the MFI of the two p24 antibodies (p24 28B7-APC and p24 KC57-PE) in the presence or absence of stimulation with PMA/ionomycin in samples from 6 untreated individuals. The MFI of p24 antibodies was measured within the p24+ gate (p24 KC57+/p24 28B7+). (TIF) [file ppat.1007619.s002.tif]

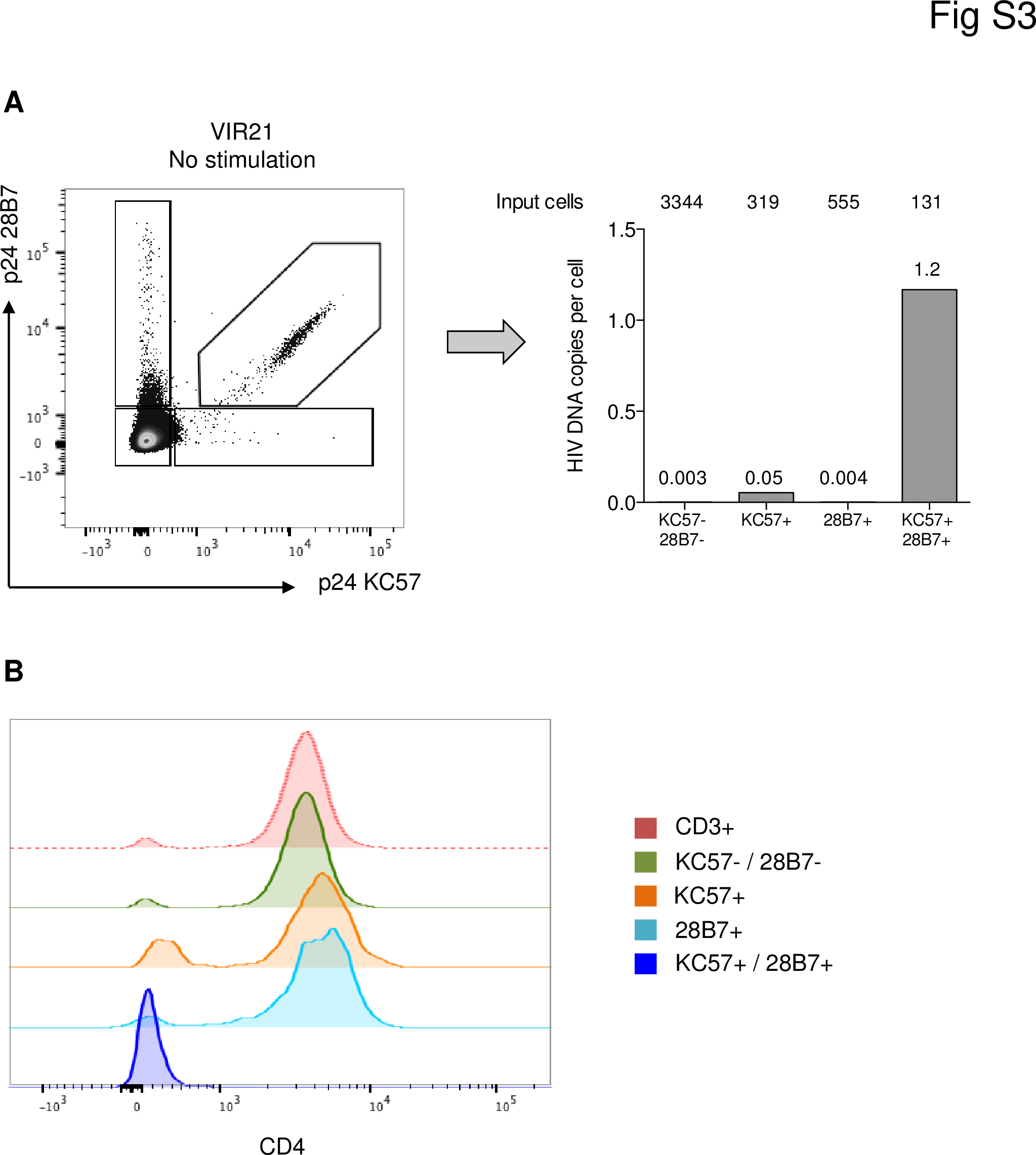

Supplement: S3 Fig — (A) Representative dot plot showing the gating strategy used to sort four populations of unstimulated cells (KC57+/28B7+, KC57+, 28B7+ and KC57-/28B7- cells) obtained from one untreated individual (VIR21). Total HIV DNA was quantified by ultrasensitive PCR in each sorted subset (right). (B) Levels of CD4 expression in the different subsets. (TIF) [file ppat.1007619.s003.tif]

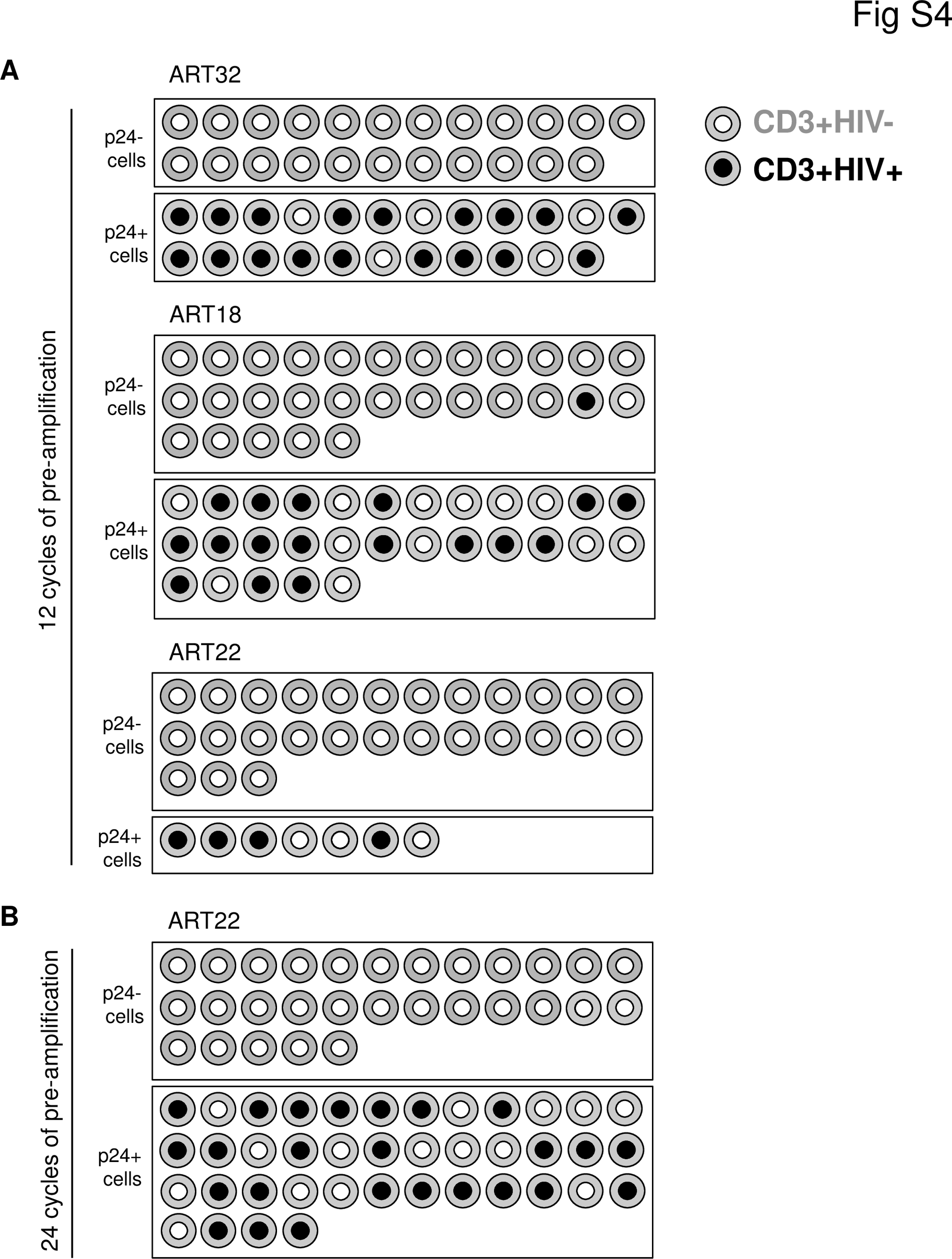

Supplement: S4 Fig — p24- and p24+ CD4 T cells from three ART-suppressed individuals were single sorted by flow cytometry and subjected to a duplex ultrasensitive PCR for the CD3 gene and the HIV genome (LTR/gag). Grey and dark circles represent successful detection of the CD3 gene and the HIV genome, respectively. A) 12 cycles of pre-PCR amplification were performed. B) 24 cycles of pre-PCR amplification were performed. (TIF) [file ppat.1007619.s004.tif]

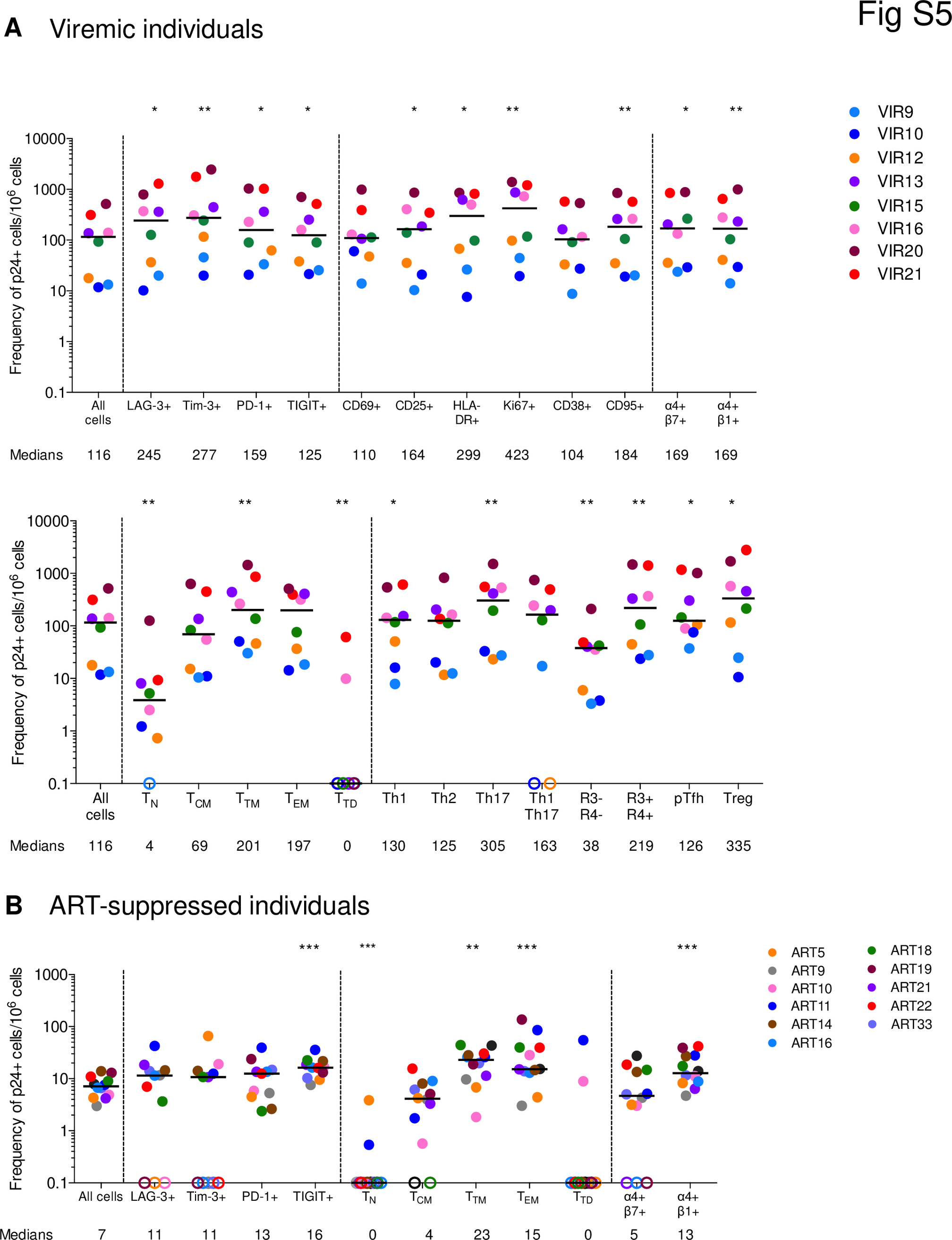

Supplement: S5 Fig — (A) Frequencies of p24+ cells in all cells and in each gated cellular subset in samples from 8 viremic individuals (same as in Figs 4 and 5). (B) Frequencies of p24+ cells in all cells and in each gated cellular subset in samples from 12 virally suppressed individuals (same as in Fig 6). Each sample is represented by a unique color-coded symbol. For statistical analyses, Wilcoxon matched-pairs signed rank test was performed: the median of each column was compared to the median of the first column (all cells). p*<0.05, p**<0.01, p***<0.001. (TIF) [file ppat.1007619.s005.tif]

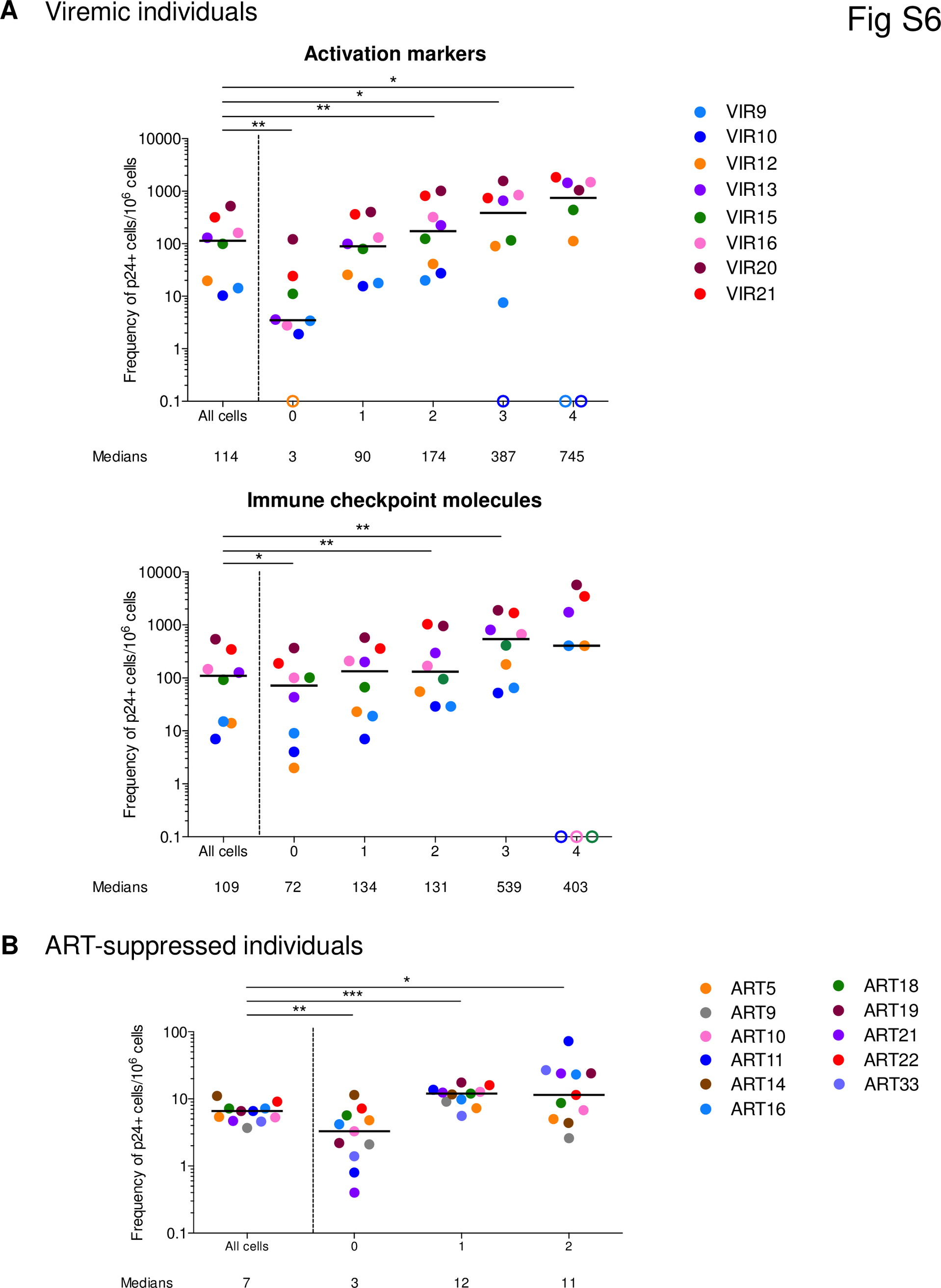

Supplement: S6 Fig — (A) Frequencies of p24+ cells in all cells and in cell subsets expressing 0, 1, 2, 3 or 4 markers in samples from 8 viremic individuals (same as in Figs 4 and 5). Analyses were performed on cells expressing CD25/CD95/HLA-DR/Ki-67 (top panel) and PD-1/TIGIT/LAG-3/Tim-3 (middle panel). (B) Frequencies of p24+ cells in all cells and in cell subsets expressing 0, 1 or 2 immune checkpoint molecules (PD-1/TIGIT) in samples from 11 virally suppressed individuals (same as in Fig 6). Each sample is represented by a unique color-coded symbol. For statistical analyses, Wilcoxon matched-pairs signed rank test was performed: the median of each column was compared to the median of the first column (all cells). p*<0.05, p**<0.01, p***<0.001. (TIF) [file ppat.1007619.s006.tif]

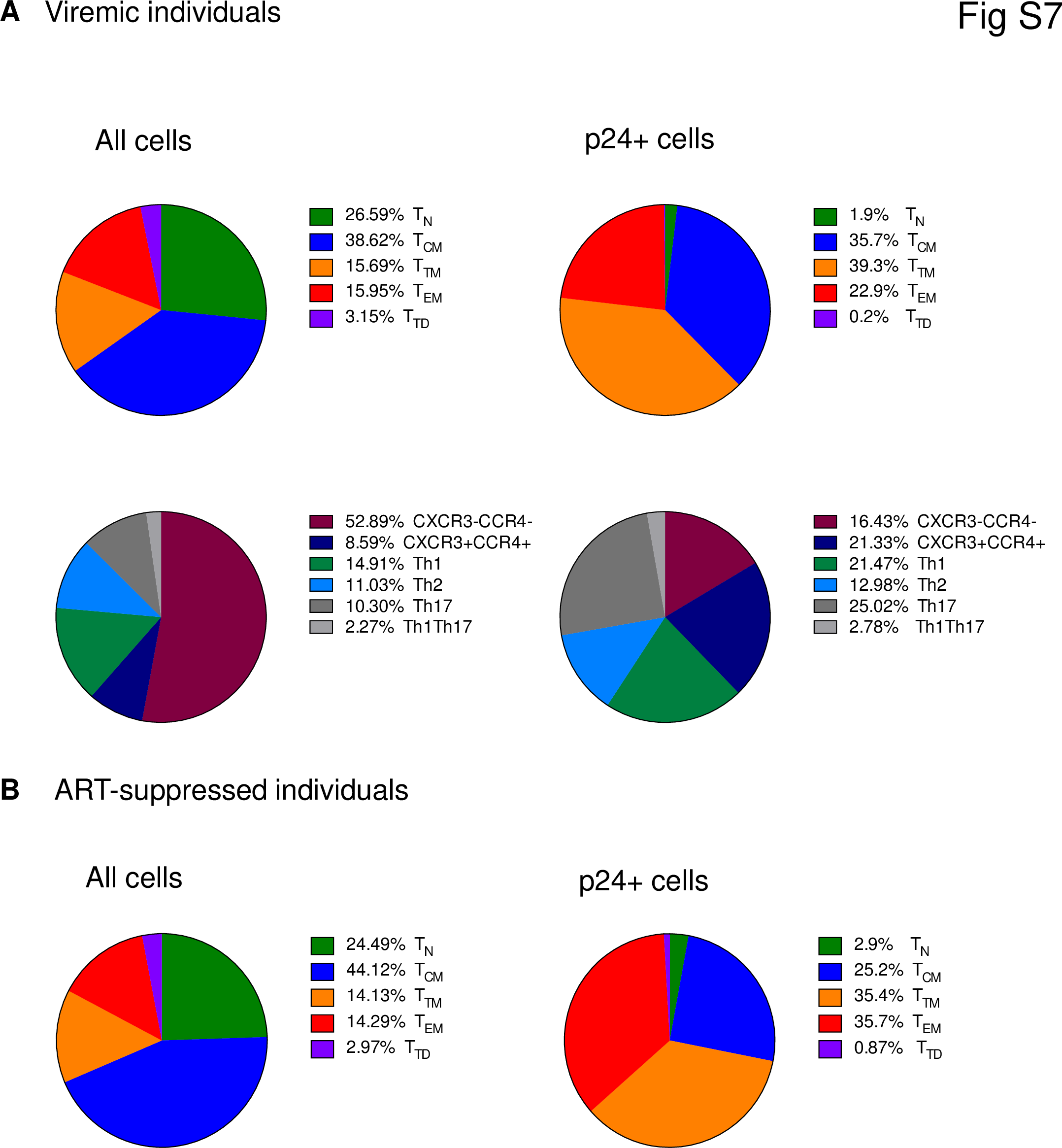

Supplement: S7 Fig — (A) Pie charts comparing the relative contributions of different subsets to the total pool of CD4 T cells (all cells, left) and to the pool of p24+ cells (right) in samples from viremic individuals. Contributions of memory subsets and effector subsets are represented. (B) Pie charts comparing the relative contributions of different subsets to the total pool of CD4 T cells (all cells, left) and to the pool of p24+ cells (right) in samples from ART-suppressed individuals. Contributions of memory subsets are represented. (TIF) [file ppat.1007619.s007.tif]

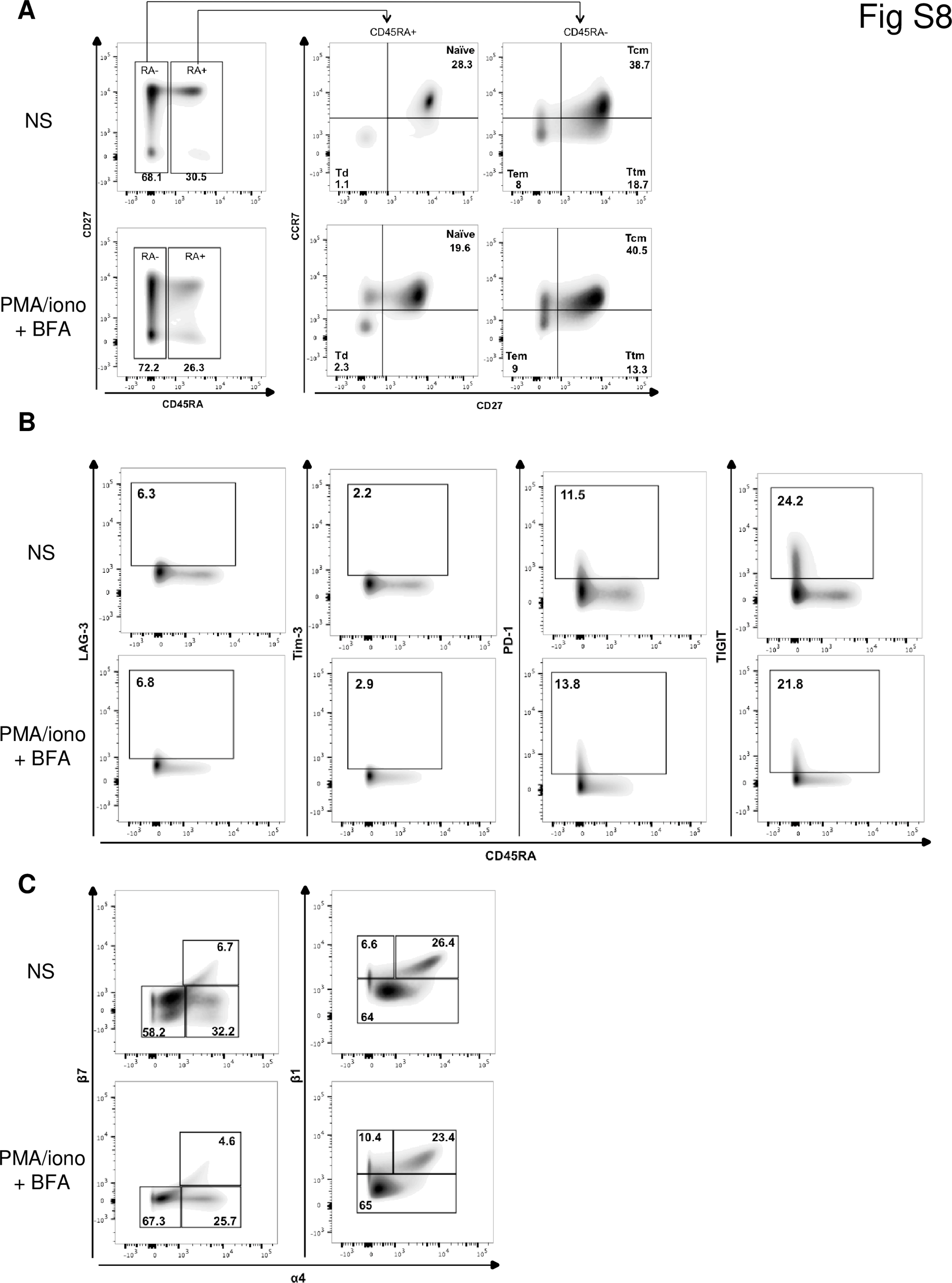

Supplement: S8 Fig — (A) Representative dot plots showing the distribution of memory CD4 T cell subsets after 24h of resting or after 24h of stimulation with PMA/ionomycin + BFA in one representative ART-suppressed individual. (B) As in A) for LAG-3, Tim-3, PD-1 and TIGIT. (C) As in A) for α4β7 and α4β1. (TIF) [file ppat.1007619.s008.tif]

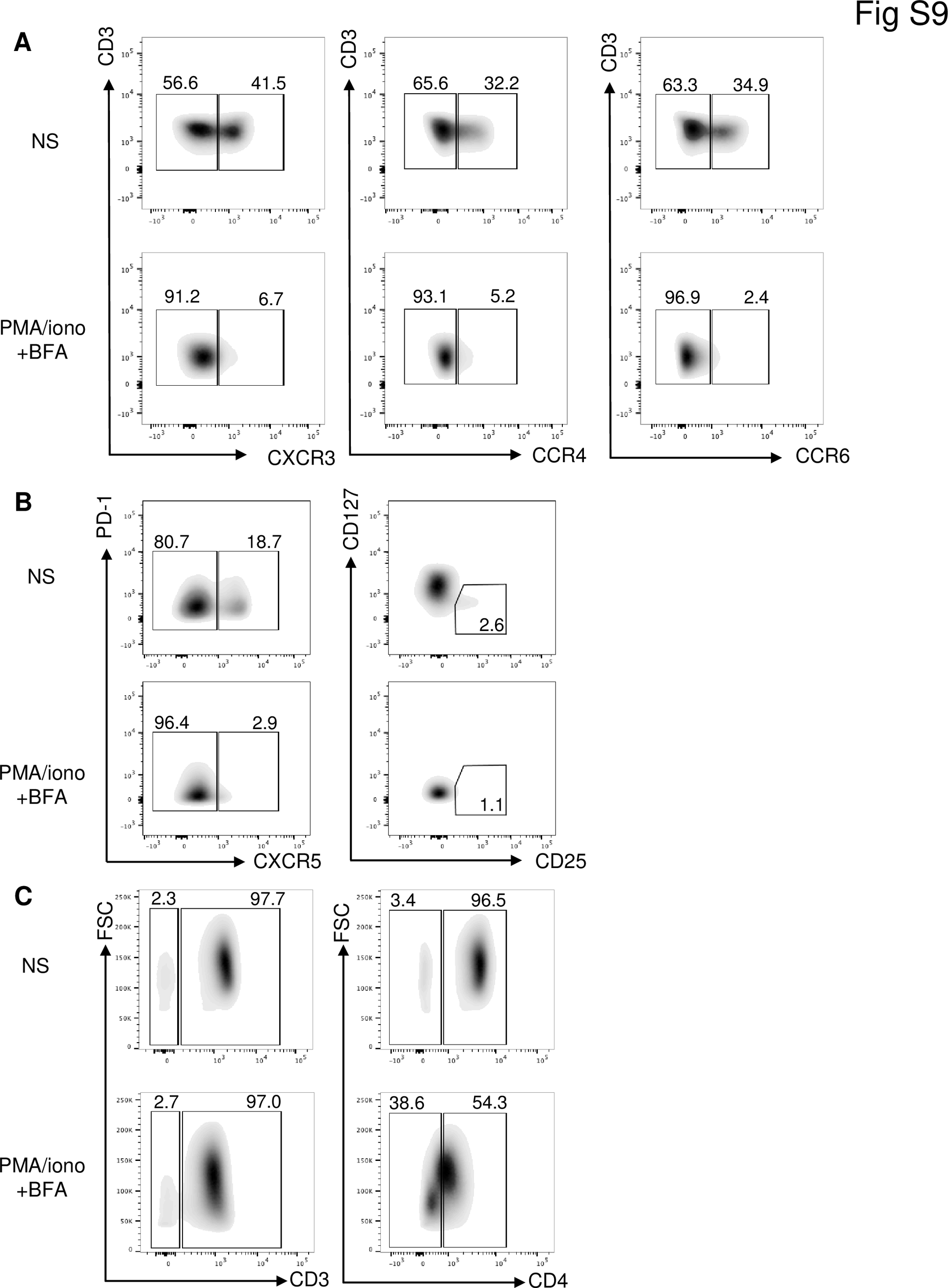

Supplement: S9 Fig — (A) Representative dot plots showing the levels of expression of CXCR3/CCR4/CCR6 after 24h of resting or after 24h of stimulation with PMA/ionomycin + BFA in one representative ART-suppressed individual. (B) As in A) for CXCR5 and CD25. (C) As in A) for CD3 and CD4. Of note, the MFI of CD3 decreased after stimulation but the frequency of CD3+ cells remained unchanged. (TIF) [file ppat.1007619.s009.tif]

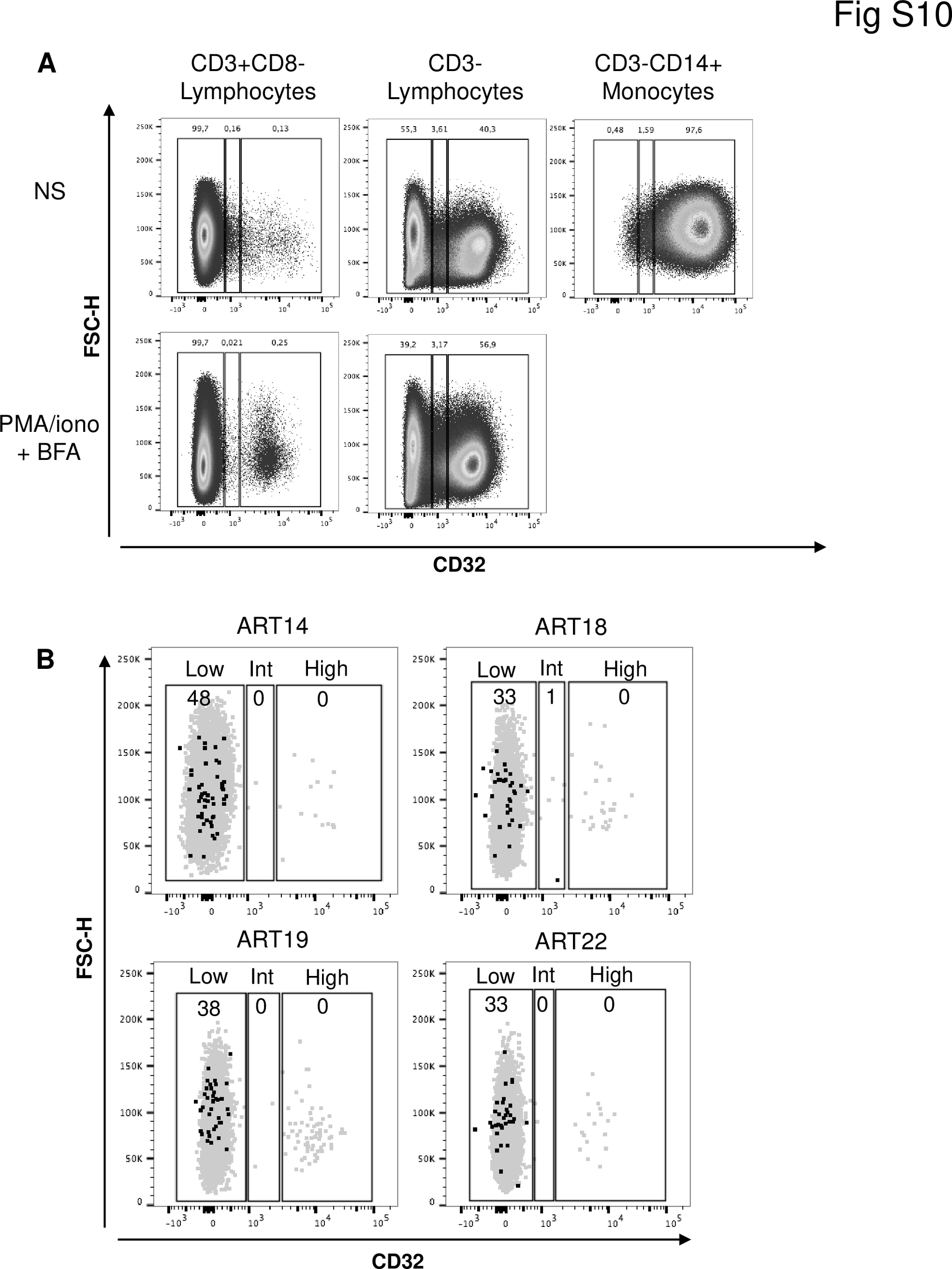

Supplement: S10 Fig — Cryopreserved PBMCs from 4 ART-suppressed individuals were stimulated with PMA/ionomycin + BFA for 24h. (A) Representative dot plots of the CD32 staining in gated CD3+CD8- lymphocytes, CD3- lymphocytes and CD3-CD14+ monocytes, in the absence of stimulation (NS) and after PMA/ionomycin stimulation. (B) Dot plots showing CD32 expression in p24+ events (black) and in all cells (grey). (TIF) [file ppat.1007619.s010.tif]

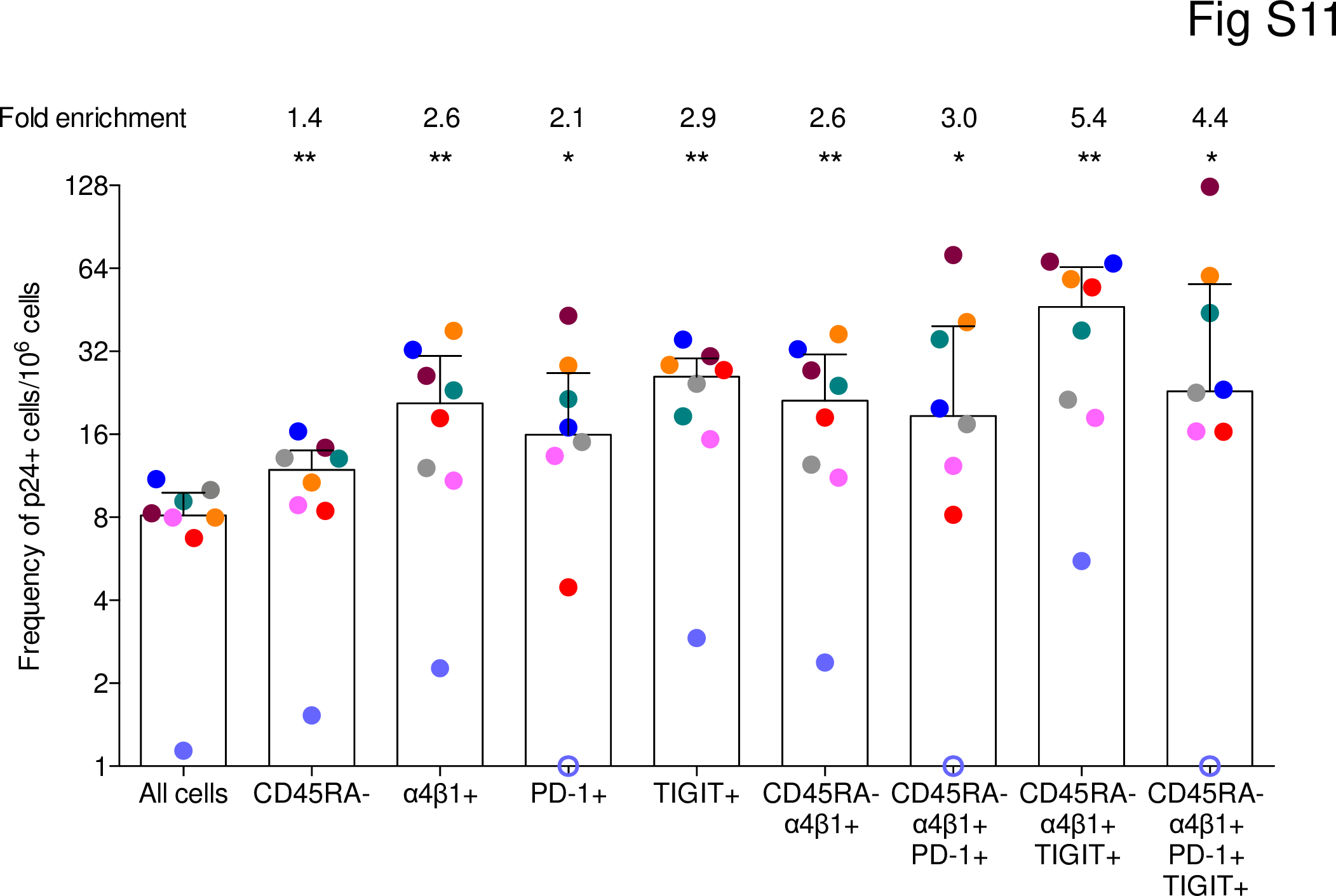

Supplement: S11 Fig — Frequencies of p24+ cells in all cells and in each gated cellular subset in samples from 8 ART-suppressed individuals. Each sample is represented by a unique color-coded symbol. Undetectable measurements are represented as open symbols. Mean folds-enrichment compared to all cells are indicated at the top of each bar. For statistical analyses, Wilcoxon matched-pairs signed rank test was performed: median values of each column were compared to the median of the first column (all cells). p*<0.05, p**<0.01. (TIF) [file ppat.1007619.s011.tif]

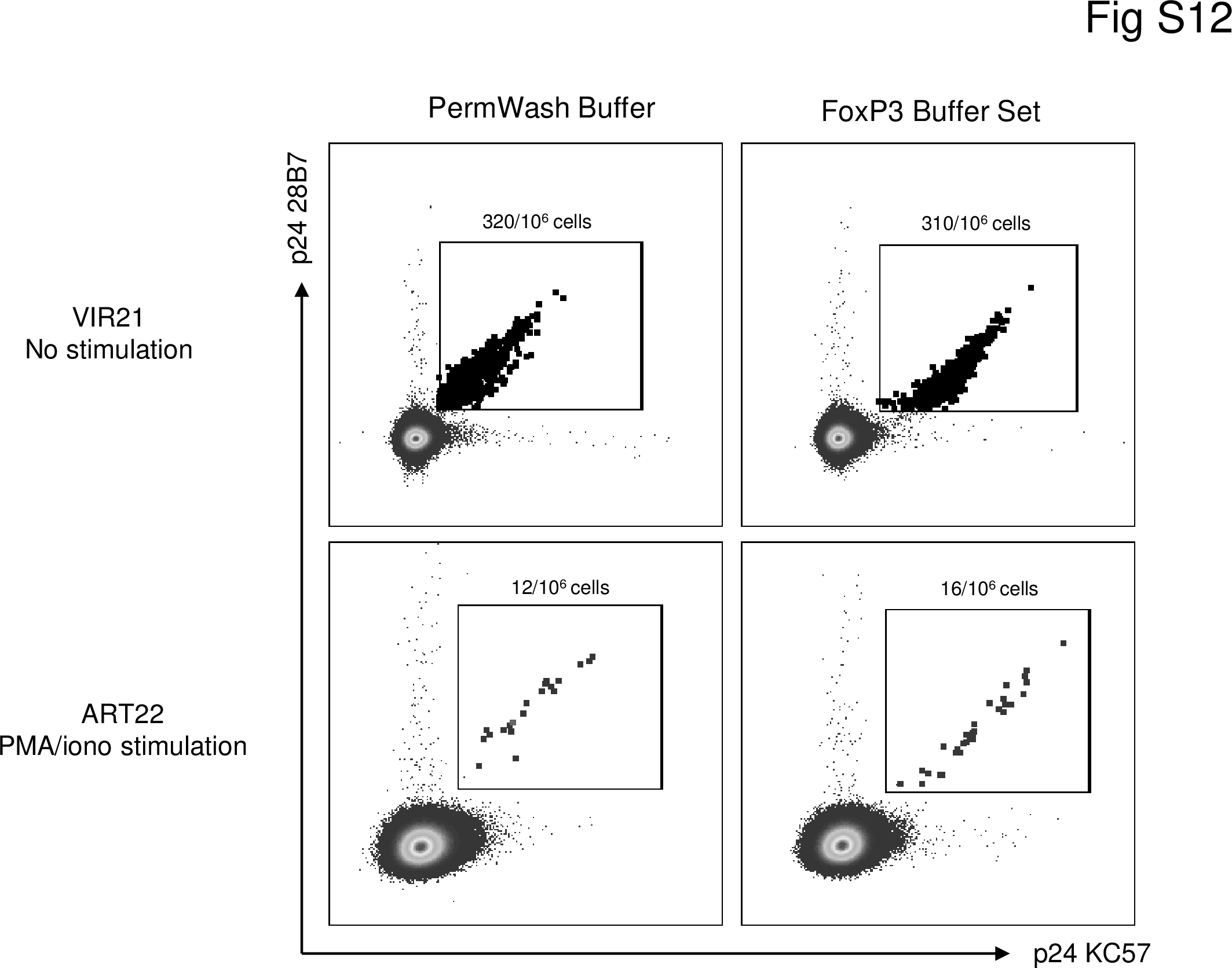

Supplement: S12 Fig — Dot plots showing the detection of p24+ cells in 2 samples, using 2 experimental conditions. Purified CD4 T cells from a viremic individual were rested for 18 hours, while purified CD4 T cells from an ART-suppressed individual were stimulated with PMA/ionomycin for 24h. The permeabilization step was performed either with the PermWash Buffer (BD) or with the FoxP3/Transcription Factor Staining Buffer Set (eBioscience). (TIF) [file ppat.1007619.s012.tif]

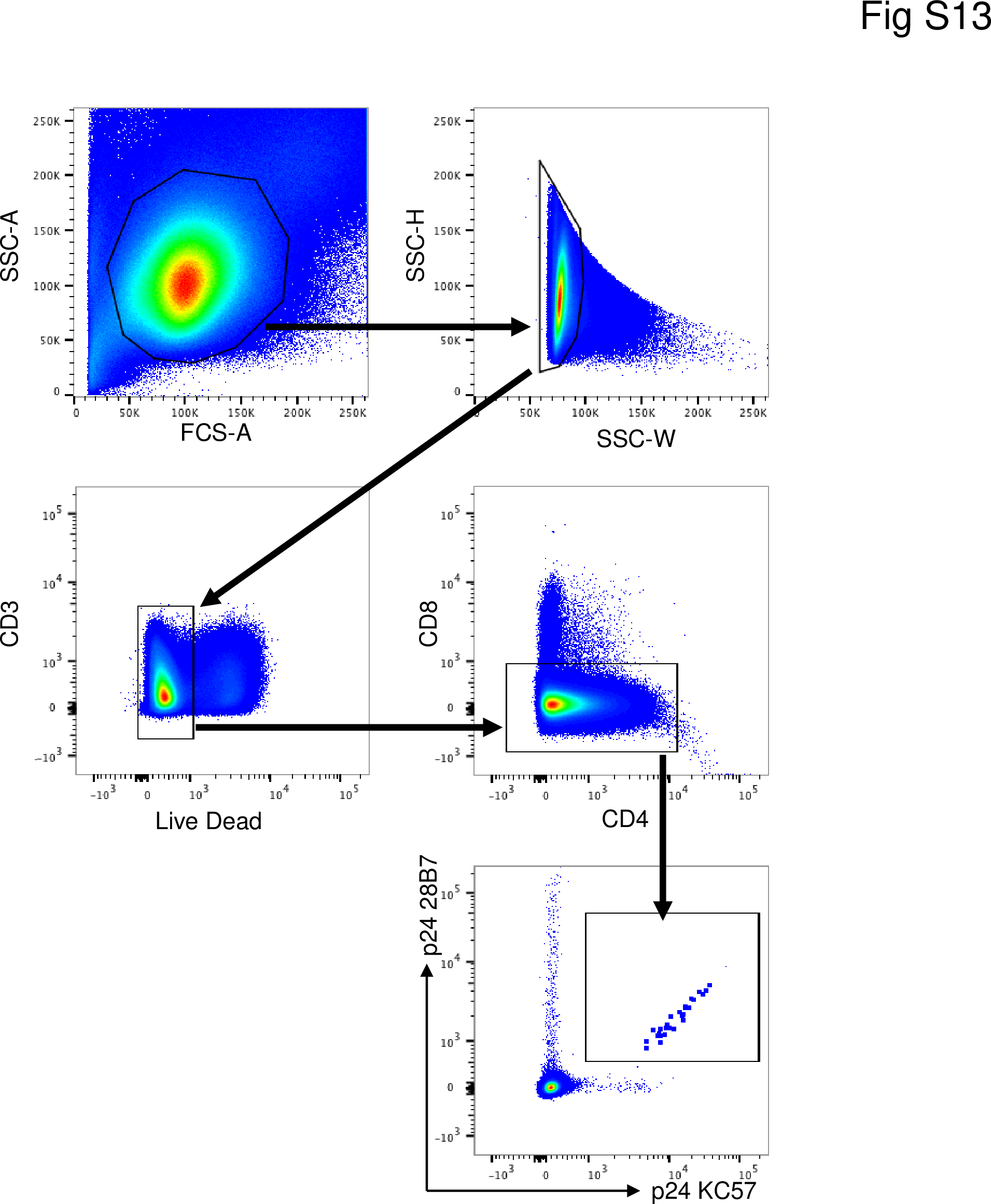

Supplement: S13 Fig — Example of the gating strategy used for a representative sample following PMA/ionomycin stimulation of CD4+ T cells obtained from an ART-suppressed individual. (TIF) [file ppat.1007619.s013.tif]
