# Supplementary material for: Single-cell characterization and quantification of translation-competent viral reservoirs in treated and untreated HIV infection
Source: PLoS Pathog. 2019 Feb 27;15(2):e1007619. doi: 10.1371/journal.ppat.1007619 (PMC6411230; doi:10.1371/journal.ppat.1007619)
Supplement: S1 Table — (DOCX) [file ppat.1007619.s014.docx]

| **ID** | **Total HIV DNA**  (HIV copies/10^6^ CD4 T cells) | **Integrated HIV DNA**  (HIV copies/10^6^ CD4 T cells) | **TILDA**  (msRNA+ cells/10^6^ CD4 T cells) | **HIV-Flow**  (p24+ cells/10^6^ cells) | **mQVOA**  (infectious units/10^6^ cells) |
| --- | --- | --- | --- | --- | --- |
| **ART1** | 173 | 125 | 12.6 | 0.5 | 0.8 |
| **ART2** | 971 | 504 | 28.8 | 4.0 | 0.9 |
| **ART3** | 1025 | 538 | 77.0 | 6.8 | 1.1 |
| **ART4** | 1335 | 1388 | 22.2 | <1.0 | 19.6 |
| **ART5** | 2397 | 1335 | 55.3 | 8.5 | 1.4 |
| **ART6** | 1438 | 515 | <1.4 | 4.7 | 1.3 |
| **ART7** | 377 | 171 | 18.0 | 2.8 | 3.2 |
| **ART8** | 1464 | 518 | 11.4 | 1.6 | 0.3 |
| **ART9** | 511 | 101 | 13.2 | 6.5 | 0.1 |
| **ART10** | 1003 | 318 | 33.6 | 5.8 | 1.4 |
| **ART11** | 1095 | 432 | 23.4 | 7.8 | 2.0 |
| **ART12** | 54 | 14 | 4.0 | <1.3 | 0.4 |
| **ART13** | 2598 | 1445 | 229.6 | 6.8 | 3.1 |
| **ART14** | 1381 | 724 | 156.2 | 20.4 | 1.4 |
| **ART15** | 1294 | 547 | 14.6 | 2.1 | 0.8 |
| **ART16** | 1035 | 895 | 33.2 | 9.0 | 0.3 |
| **ART17** | 512 | 322 | 6.6 | <0.3 | 0.4 |
| **ART18** | 537 | 829 | 90.0 | 12.0 | 0.9 |
| **ART19** | 1416 | 1058 | 88.6 | 12.1 | 46.9 |
| **ART20** | 244 | 167 | 29.2 | 0.2 | <0.08 |
| **ART21** | 98 | 43 | 5.6 | 3.9 | 0.3 |
| **ART22** | 2386 | 1607 | 12.7 | 11.9 | 2.0 |
| **ART23** | 89 | 95 | 2.8 | 0.7 | <0.08 |
| **ART24** | 108 | 109 | 1.5 | 0.3 | 0.3 |
| *Median* | 1014 | 510 | 20.1 | 4.3 | 0.9 |
| *IQ range* | *[344-1390]* | *[156-846]* | *[10.2-39.0]* | *[0.7-8.0]* | *[0.3-1.6]* |

**Table S1 : Frequencies of infected cells measured by different assays in samples from ART-suppressed individuals**
