# Supplementary material for: Single-cell characterization and quantification of translation-competent viral reservoirs in treated and untreated HIV infection
Source: PLoS Pathog. 2019 Feb 27;15(2):e1007619. doi: 10.1371/journal.ppat.1007619 (PMC6411230; doi:10.1371/journal.ppat.1007619)
Supplement: S2 Table — (DOCX) [file ppat.1007619.s015.docx]

**Table S2: Panels of antibodies used for phenotyping of p24+ cells**

| ***Samples from viremic individuals*** | | |  |  |  |  |
| --- | --- | --- | --- | --- | --- | --- |
|  | **Antibody** | **Clone** | | **Colour** | **Company** | **Catalog number** |
| Panel in Fig. 4A | CD3 | UCHT1 | | A700 | BD | 557943 |
|  | CD4 | SK3 | | BUV496 | BD | 564651 |
|  | CD45RA | HI100 | | BV786 | BD | 563870 |
|  | CD69 | FN50 | | BUV737 | BD | 564439 |
|  | CD25 | M-A251 | | BV421 | BD | 562442 |
|  | HLA-DR | G46-6 | | BUV395 | BD | 564040 |
|  | CD38 | HIT2 | | PerCPCy5.5 | BD | 551400 |
|  | Ki67 | MOPC-21 | | FITC | BD | 556026 |
|  | CD95 | DX2 | | Pe-Cy7 | BD | 561633 |
| Panel in Fig. 4B | CD3 | UCHT1 | | BUV395 | BD | 557943 |
|  | CD4 | SK3 | | BUV496 | BD | 564651 |
|  | CD45RA | HI100 | | A700 | BD | 560673 |
|  | PD-1 | EH12.1 | | Pe-Cy7 | BD | 561272 |
|  | LAG-3 | Polyclonal | | FITC | R&D | FAB2319F |
|  | TIGIT | MBSA43 | | PerCPEF710 | eBioscience | 46-9500-41 |
|  | Tim-3 | F38-2E2 | | BV421 | BioLegend | 345008 |
| Panel in Fig. 5A and Fig. 5C | CD3 | UCHT1 | | BUV395 | BD | 557943 |
|  | CD4 | SK3 | | BUV496 | BD | 564651 |
|  | CD45RA | HI100 | | BV786 | BD | 563870 |
|  | CCR7 | 3D12 | | BB700 | BD | 566437 |
|  | CD27 | O353 | | BV421 | BioLegend | 302823 |
|  | CXCR5 | RF8B2 | | BB515 | BD | 564624 |
|  | PD-1 | EH12.1 | | BUV737 | BD | 565299 |
|  | CD127 | HIL-7R-M21 | | BV605 | BD | 562662 |
|  | CD25 | M-A251 | | A700 | BD | 561398 |
| Panel in Fig. 5B and Fig. 5D | CD3 | UCHT1 | | A700 | BD | 557943 |
|  | CD4 | SK3 | | BUV496 | BD | 564651 |
|  | CXCR3 | 1C6/CXCR3 | | PerCP-Cy5.5 | BD | 560832 |
|  | CCR4 | 1G1 | | BV421 | BD | 562579 |
|  | CCR6 | 11A9 | | BUV737 | BD | 564377 |
|  | Alpha4 | 9F10 | | Pe-Cy7 | BioLegend | 304313 |
|  | Beta1 | MAR4 | | BB515 | BD | 564565 |
|  | Beta7 | FIB504 | | BUV395 | BD | 744014 |
| ***Samples from ART-suppressed individuals*** | | | |  |  |  |
|  | **Antibody** | **Clone** | | **Colour** | **Company** | **Catalog number** |
| Panel in Fig. 6A | CD3 | UCHT1 | | BUV395 | BD | 557943 |
|  | CD4 | SK3 | | BUV496 | BD | 564651 |
|  | CD45RA | HI100 | | BV786 | BD | 563870 |
|  | PD-1 | EH12.1 | | BUV737 | BD | 565299 |
|  | LAG-3 | Polyclonal | | FITC | R&D | FAB2319F |
|  | TIGIT | MBSA43 | | PerCP-eF710 | eBioscience | 46-9500-41 |
|  | Tim-3 | F38-2E2 | | BV421 | BioLegend | 345008 |
| Panel in Fig. 6B | CD3 | UCHT1 | | BUV395 | BD | 557943 |
|  | CD4 | SK3 | | BUV496 | BD | 564651 |
|  | CD45RA | HI100 | | BV786 | BD | 563870 |
|  | CCR7 | 3D12 | | BB700 | BD | 566437 |
|  | CD27 | O353 | | BV421 | BioLegend | 302823 |
| Panel in Fig. 6C | CD3 | UCHT1 | | A700 | BD | 557943 |
|  | CD4 | SK3 | | BUV496 | BD | 564651 |
|  | Alpha4 | 9F10 | | Pe-Cy7 | BioLegend | 304313 |
|  | Beta1 | MAR4 | | BB515 | BD | 564565 |
|  | Beta7 | FIB504 | | BUV395 | BD | 744014 |
