# Supplementary material for: Single-cell characterization and quantification of translation-competent viral reservoirs in treated and untreated HIV infection
Source: PLoS Pathog. 2019 Feb 27;15(2):e1007619. doi: 10.1371/journal.ppat.1007619 (PMC6411230; doi:10.1371/journal.ppat.1007619)
Supplement: S3 Table — (DOCX) [file ppat.1007619.s016.docx]

**Table S3: Median fold differences in the frequencies of p24+ cells and all cells expressing a given cellular marker.**

| **Viremic individuals** | | |
| --- | --- | --- |
| Fig. 4A | CD69+ | 1.19 |
|  | CD25+ | 1.55 |
|  | HLA-DR+ | 2.21 |
|  | Ki67+ | 3.45 |
|  | CD38+ | 1.15 |
|  | CD95+ | 1.72 |
| Fig. 4B | LAG-3+ | 2.02 |
|  | Tim-3+ | 3.28 |
|  | PD-1+ | 2.49 |
|  | TIGIT+ | 1.59 |
| Fig. 5A | CD45RA- | 1.48 |
|  | TN | 0.04 |
|  | TCM | 0.85 |
|  | TTM | 2.60 |
|  | TEM | 1.38 |
|  | TTD | 0.00 |
| Fig. 5B | CXCR3-CCR4- | 0.30 |
|  | CXCR3+CCR4+ | 2.53 |
|  | Th1 | 1.15 |
|  | Th1Th17 | 1.50 |
|  | Th2 | 1.17 |
|  | Th17 | 2.54 |
| Fig. 5C | pTfh | 2.53 |
|  | Treg | 3.13 |
| Fig 5D | α4β7 | 2.07 |
|  | α4β1 | 2.01 |

| **ART-suppressed individuals** | | |
| --- | --- | --- |
| Fig. 6A | LAG-3+ | 1.30 |
|  | Tim-3+ | 1.27 |
|  | PD-1+ | 1.65 |
|  | TIGIT+ | 2.23 |
| Fig. 6B | CD45RA- | 1.31 |
|  | TN | 0.00 |
|  | TCM | 0.67 |
|  | TTM | 2.63 |
|  | TEM | 2.55 |
|  | TTD | 0.00 |
| Fig. 6C | α4β7 | 0.78 |
|  | α4β1 | 2.21 |
